# PPARγ signaling pathway: strategies and limitations of natural compounds in the treatment of ulcerative colitis

**DOI:** 10.3389/fphar.2026.1977683

**Published:** 2026-10-20

**Authors:** Licheng Liang, Hualiang Deng

**Affiliations:** 1 Shandong University of Traditional Chinese Medicine, Jinan, China; 2 Affiliated Hospital of Shandong University of Traditional Chinese Medicine, Jinan, China

**Keywords:** intestinal inflammation, intestinal mucosal barrier, natural compounds, PPARγ, ulcerative colitis

## Abstract

Ulcerative colitis (UC) is a chronic, relapsing inflammatory bowel disease characterized by persistent inflammatory activation, immune dysregulation, gut microbiota dysbiosis, and damage to the mucosal barrier. Peroxisome proliferator-activated receptor gamma (PPARγ) is a key nuclear receptor involved in maintaining intestinal immunity and mucosal homeostasis. Reduced PPARγ expression or impaired function may weaken its negative regulatory role in pro-inflammatory signaling and disrupt the Th17/Treg balance, macrophage polarization, gut microbiota homeostasis, and barrier integrity, making PPARγ a crucial molecular hub linking multiple pathological processes in UC. Due to their structural diversity and multi-target regulatory properties, natural compounds are attracting increasing attention as potential modulators of PPARγ-related networks. This review systematically summarizes the current preclinical evidence regarding natural compounds that target PPARγ and its associated signaling networks in UC, with particular emphasis on their roles in regulating inflammatory responses, immune cell function, the Th17/Treg balance, the gut microbiota, and mucosal barrier integrity through PPARγ-related pathways (including AMPK, NF-κB, mTOR, SIRT1, MAPK, Nrf2, and STAT). Importantly, the currently available evidence primarily stems from *in vitro* cellular and *in vivo* animal studies; therefore, it mainly provides mechanistic and preclinical support rather than evidence of clinical efficacy. Current research is also limited by insufficient evaluation of specific targets, reliance on a limited range of disease models, and inadequate characterization of pharmacokinetic and safety profiles. Future research should prioritize rigorous evaluation of PPARγ-dependent mechanisms, conduct pharmacokinetic and safety assessments, and design rigorous human studies and clinical trials to establish the therapeutic potential of these natural compounds in UC. This review provides a framework for critically evaluating the existing evidence and guiding the development and evaluation of PPARγ-targeting natural compounds for UC.

## 1 Introduction

Ulcerative colitis (UC) is a chronic, recurrent inflammatory bowel disease (IBD) that primarily affects the mucosa of the colon and rectum. Lesions typically originate in the rectum and spread continuously toward the proximal colon; clinically, the disease is characterized by recurrent abdominal pain, diarrhea, mucus and pus-containing bloody stools, and tenesmus (Rubin et al., 2025; Le Berre et al., 2023). In recent years, the global burden of UC has continued to rise, with the disease rapidly spreading from traditional high-prevalence regions in Europe and the United States to newly industrialized countries, making it a global chronic gastrointestinal disease that cannot be ignored. Long-term, persistent mucosal inflammation not only leads to recurrent ulcer formation, disruption of the intestinal mucosal barrier, and intestinal dysfunction, but also significantly increases the risk of long-term malignant complications such as colorectal cancer (Zhang et al., 2023). It is currently widely accepted in the scientific community that UC is not a disease driven by a single factor, but rather the result of the interplay and combined effects of multiple pathological mechanisms, including genetic susceptibility, environmental exposures, gut microbiota dysbiosis, damage to the intestinal mucosal barrier, and abnormal immune responses. These factors form a complex local network of inflammation, immunity, microbiota, and barrier dysfunction within the intestinal mucosa, resulting in highly heterogeneous pathogenesis of UC and posing significant challenges for clinical treatment (Wangchuk et al., 2024; Su et al., 2024). Therefore, in-depth investigation of the core molecular nodes capable of simultaneously coordinating these multiple pathological processes holds important theoretical value and clinical significance for elucidating the pathogenesis of UC and developing novel therapeutic strategies.

Current clinical treatment regimens for UC primarily include aminosalicylate preparations, glucocorticoids, immunosuppressants, and biologics. Although these medications play a crucial role in inducing remission and maintenance therapy, there remain numerous unmet clinical needs (Yang and Zhang, 2025). Aminosalicylic acid-based drugs are suitable for patients with mild to moderate disease but have limited efficacy in the treatment of moderate to severe active disease; long-term use of glucocorticoids can lead to serious adverse reactions such as osteoporosis, infections, and metabolic disorders (De Deo et al., 2024). Immunosuppressants take time to take effect and carry risks such as bone marrow suppression and liver and kidney damage; although biologics have significantly improved the prognosis of moderate-to-severe UC, a considerable proportion of patients still experience primary or secondary non-response, and the high cost of treatment limits their widespread use (Kazemifard et al., 2025). More critically, the aforementioned treatment approaches primarily focus on inhibiting a single inflammatory pathway, making it difficult to simultaneously correct the coexisting immune imbalance, microbiota dysbiosis, and barrier disruption in UC; consequently, achieving long-term mucosal healing and sustained remission remains challenging (Liang et al., 2024). Against this backdrop, the search for novel therapeutic targets that can act on multiple pathological pathways and combine anti-inflammatory and tissue-repair functions has become a hot topic in UC research. Due to their dual characteristics of transcriptional regulation and ligand-targeted specificity, nuclear receptors have gradually emerged as important candidate targets for drug development in inflammatory diseases.

Although the pathogenesis of UC has not yet been fully elucidated, current research suggests that its onset and progression result from the interaction of multiple factors, including genetic susceptibility, environmental factors, gut microbiota dysbiosis, mucosal barrier dysfunction, and abnormalities in the innate and adaptive immune systems. These pathological changes are interlinked and collectively maintain a state of persistent inflammation in the intestinal mucosa (Hanzel et al., 2022). Within this complex regulatory network, peroxisome proliferator-activated receptor γ (PPARγ)-a key nuclear receptor that maintains colonic immune and mucosal homeostasis—is believed to be closely associated with the persistence of UC inflammation and disease progression when its expression is reduced or its transcriptional activity is impaired (Fang et al., 2021). Mechanistically, impaired PPARγ function can lift its negative regulation of pro-inflammatory transcriptional programs such as NF-κB, AP-1, and STAT, promoting the sustained expression of inflammatory mediators such as TNF-α, IL-1β, IL-6, and iNOS, thereby amplifying local inflammatory signals and weakening mucosal immune tolerance; At the same time, abnormal PPARγ signaling can also affect Th17/Treg immune homeostasis and macrophage phenotypic conversion, and interact with gut microbiota metabolic dysregulation and mucosal barrier damage, further promoting inflammatory cell infiltration, disruption of epithelial structure, and impaired mucosal repair (Decara et al., 2020). Therefore, PPARγ dysregulation is not limited to a single inflammatory pathway but may form a regulatory network that continuously amplifies pathological damage in UC by synergistically influencing inflammatory responses, immune homeostasis, the gut microbiome, and mucosal barrier function, making it a key molecular hub linking the multiple pathological processes of UC.

Natural compounds are attracting increasing attention in research on the prevention and treatment of UC due to their widespread sources, structural diversity, multi-target action, and relatively low toxicity (Yue et al., 2026). A growing body of evidence suggests that various naturally derived bioactive compounds—including flavonoids, triterpenes, alkaloids, polysaccharides, and phenolic compounds—can exert anti-inflammatory and mucosal protective effects in the gut by activating the PPARγ signaling pathway. These natural compounds can, through PPARγ, further regulate multiple downstream signaling networks such as NF-κB, AMPK, mTOR, SIRT1, MAPK, Nrf2, and STAT, among others, to synergistically intervene in the pathological progression of UC at multiple levels—including inhibiting inflammatory transcriptional programs, reshaping immune cell polarization, restoring the Th17/Treg balance, improving gut microbiota metabolism, enhancing intestinal mucosal barrier integrity, and alleviating oxidative stress. However, existing studies still have significant shortcomings in terms of the specificity of target evaluation, limitations of animal models, lack of pharmacokinetic data, and insufficient evidence for clinical translation. Based on this, this review systematically summarizes the multifaceted regulatory roles of PPARγ in the pathogenesis of UC, synthesizes research progress on the use of natural compounds to intervene in UC via PPARγ-related signaling pathways, and conducts an in-depth analysis of the strategic advantages and translational limitations of current research, with the aim of providing guidance for the development of novel therapeutic agents and mechanistic studies for UC.

## 2 Overview of PPARγ

### 2.1 Molecular structure, subtypes, and transcriptional regulatory characteristics of PPARγ

PPARγ is an important member of the ligand-dependent nuclear receptor superfamily and is encoded by the PPARG gene located on human chromosome 3p25. The PPARγ protein possesses a typical nuclear receptor structure, primarily consisting of the N-terminal A/B regions, the DNA-binding domain (DBD), the hinge region, and the C-terminal ligand-binding domain (LBD) (Chen et al., 2023). The N-terminal A/B region contains the ligand-independent transcriptional activation domain AF-1, which can be regulated by post-translational modifications such as phosphorylation; the highly conserved DBD contains two zinc-finger structures responsible for recognizing PPAR response elements (PPREs) in the promoter or enhancer regions of target genes; the hinge region confers structural flexibility to the receptor and participates in nuclear localization; the C-terminal LBD contains the ligand-binding pocket and the ligand-dependent transcriptional activation domain AF-2, which serves as the primary structural basis for the action of ligands such as fatty acid derivatives, prostaglandin metabolites, and thiazolidinedione drugs (Qian et al., 2024). Upon ligand binding, PPARγ undergoes a conformational change and forms a heterodimer with the retinoid X receptor (RXR), subsequently binding to PPRE to regulate target gene transcription by recruiting coactivators or releasing co-repressors (Li et al., 2022). In addition, PPARγ can also intervene in inflammation-related transcriptional programs such as NF-κB, AP-1, and STAT through transcriptional repression mechanisms, thereby establishing an anti-inflammatory action pattern distinct from classical PPRE-dependent transcriptional regulation (Carvalho et al., 2021). PPARγ has multiple transcripts formed by different promoters and alternative splicing; among these, the protein subtypes with clear functional significance primarily include PPARγ1 and PPARγ2. PPARγ1 is widely distributed in adipose tissue, colonic epithelium, and various cell types such as macrophages, dendritic cells, and lymphocytes, whereas PPARγ2 has an N-terminus that is approximately 30 amino acids longer than that of PPARγ1; it is primarily enriched in adipose tissue and exhibits strong transcriptional activity in lipogenesis (Chang and Ha, 2018).

### 2.2 Physiological functions of PPARγ and its pathological role in chronic inflammatory diseases

From a physiological perspective, PPARγ was initially regarded as a central regulator of adipocyte differentiation and lipid storage, serving as a key molecular hub linking metabolic homeostasis, immune regulation, and tissue homeostasis. PPARγ activation promotes adipocyte differentiation, fatty acid uptake, and storage; enhances insulin sensitivity; and contributes to the maintenance of glucose and lipid metabolic homeostasis (Janani and Ranjitha Kumari, 2015). In the immune system, PPARγ limits excessive immune responses by regulating macrophage function, T-cell differentiation, and the production of inflammatory mediators (Montaigne et al., 2021). In the gastrointestinal tract, PPARγ is highly expressed in the colonic mucosa, particularly in the colonic epithelium, and plays a role in maintaining mucosal immune tolerance, gut microbiota homeostasis, and barrier integrity (Byndloss et al., 2017). Therefore, PPARγ is not merely a metabolic regulatory receptor but an important nuclear transcription factor that coordinates the body’s metabolic state with inflammatory and immune responses. Furthermore, abnormalities in PPARγ expression or activity are closely associated with the onset and progression of various diseases, and its pathological significance is markedly dependent on the tissue and disease context. In metabolic diseases such as obesity, insulin resistance, and type 2 diabetes, PPARγ signaling imbalances can affect adipocyte differentiation, ectopic lipid deposition, and insulin sensitivity (Zhou Y. et al., 2023). In atherosclerosis and cardiovascular disease, PPARγ contributes to pathogenesis by regulating macrophage lipid metabolism and inflammatory phenotypes (Ivanova et al., 2015). In tumors, PPARγ may exert context-dependent effects by influencing cell differentiation, proliferation, metabolism, and the tumor immune microenvironment (Mal et al., 2021). Notably, there is a particularly close association between PPARγ and chronic inflammatory diseases. Inflammatory stimuli can reduce PPARγ expression or attenuate its transcriptional activity, while PPARγ dysfunction can lift its negative regulation of pro-inflammatory transcriptional programs, leading to a feedback loop of sustained inflammatory amplification (Bellver-Sanchis et al., 2024). Mechanistically, PPARγ inhibits the nuclear translocation and transcriptional activity of NF-κB p65 and interacts with inflammation-related signaling pathways such as AP-1 and STAT, thereby limiting the expression of TNF-α, IL-1β, IL-6, iNOS, and various chemokines; simultaneously, it influences the inflammatory phenotype of macrophages and T-cell immune responses. Consequently, reduced PPARγ expression or impaired function has been linked to the onset and progression of chronic inflammatory diseases such as atherosclerosis, nonalcoholic fatty liver disease, and IBD.

## 3 Methods and literature search strategy

This review systematically searched the PubMed, Web of Science, and China National Knowledge Infrastructure (CNKI) databases for relevant literature published between 2016 and 2026, employing a search strategy that combined Medical Subject Headings (MeSH) terms and free-text keywords, which was optimized according to the characteristics of each database. The search focused on three core themes: “PPARγ,” “ulcerative colitis (UC),” and “natural compounds.” The strategy integrated synonyms related to PPARγ (e.g.,“PPAR gamma,”“peroxisome proliferator-activated receptor γ,” “PPARG”), terms related to UC (e.g.,“ulcerative colitis,” “inflammatory bowel disease,” “colitis”), and terms related to natural products (such as “natural products,” “bioactive compounds,” “phytochemicals,” “plant-derived compounds,” “traditional Chinese medicine,” “extracts,” and keywords for major compound categories). The included studies covered natural monomeric compounds with well-defined chemical structures, metabolites of natural origin with well-defined structures, and plant or traditional Chinese medicine extracts with relatively well-defined chemical compositions and quality control information; monomeric compounds from medicinal plants that had been fully isolated, identified, and structurally characterized were included in the study scope; however, multi-component traditional Chinese medicine formulations were excluded due to their complex composition and the difficulty in accurately attributing their effects to specific active ingredients and molecular mechanisms related to PPARγ-mediated pharmacological actions. A total of 352 articles were identified during the initial screening. The quality of the included studies and the reliability of the evidence regarding the mechanisms were comprehensively assessed based on experimental design, model selection, methodological standards, completeness of results, and the degree of evaluation of PPARγ effects. Subsequently, PPARγ-related mechanisms were classified according to their level of evidence, including PPARγ expression regulation, molecular docking predictions, pharmacological blockade/rescue experiments, direct binding or transcriptional activity assays, and genetic evaluation. Ultimately, 25 preclinical studies were included for mechanism integration and critical analysis; all studies were derived from *in vitro* cell experiments and/or animal models and did not involve human clinical studies or clinical trials (Figure 1).

**FIGURE 1:**
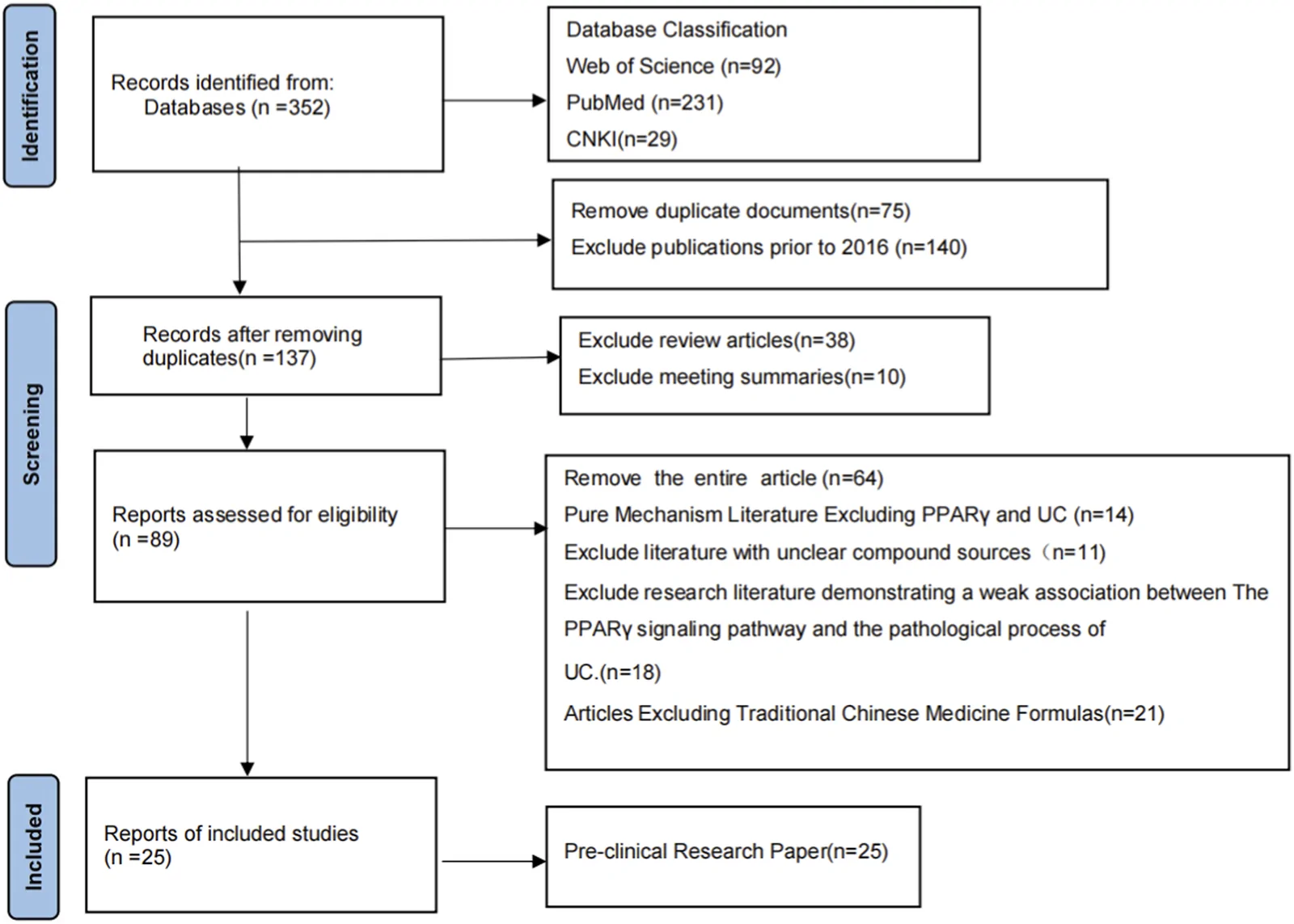
Flowchart of literature search.

## 4 The regulatory mechanism of PPARγ in UC

### 4.1 PPARγ and intestinal inflammation

PPARγ is a key nuclear receptor that regulates intestinal inflammatory responses; it is highly expressed in colonic epithelial cells and plays a role in maintaining the immune homeostasis of the intestinal mucosa. Studies have shown that PPARγ dysfunction is closely associated with inflammatory activity in UC, and its core function lies in the negative regulation of pro-inflammatory signal transduction and the transcription of inflammation-related genes (Cheng et al., 2024). Under normal physiological conditions, upon activation, PPARγ inhibits the sustained activation of pro-inflammatory signals such as NF-κB through both transcription-dependent and transcription-independent mechanisms, thereby limiting the expression of inflammatory mediators such as TNF-α, IL-1β, and IL-6 and maintaining local immune tolerance in the colon (Huang et al., 2024). Furthermore, PPARγ signaling is closely associated with the severity of mucosal inflammation in UC; impaired PPARγ function weakens its inhibitory effect on inflammatory transcriptional programs, leading to sustained amplification of pro-inflammatory signaling (Guo and Wang, 2023). In the inflammatory microenvironment of UC, microbiota-derived stimuli from the intestinal lumen can induce innate immune responses mediated by pattern recognition receptors (PRRs) such as TLRs, which in turn activate inflammatory signaling pathways including NF-κB; when PPARγ-mediated negative feedback regulation is insufficient, inflammatory signaling cannot be effectively terminated, thereby promoting the sustained release of pro-inflammatory cytokines and creating an amplifying effect on the inflammatory response. Related studies have shown that PPARγ activation can attenuate NF-κB activity in a colitis model, whereas this anti-inflammatory effect is significantly reduced when PPARγ function is inhibited, further supporting the negative regulatory role of PPARγ in the regulation of intestinal inflammation (Tian et al., 2025) (Figure 2).

**FIGURE 2:**
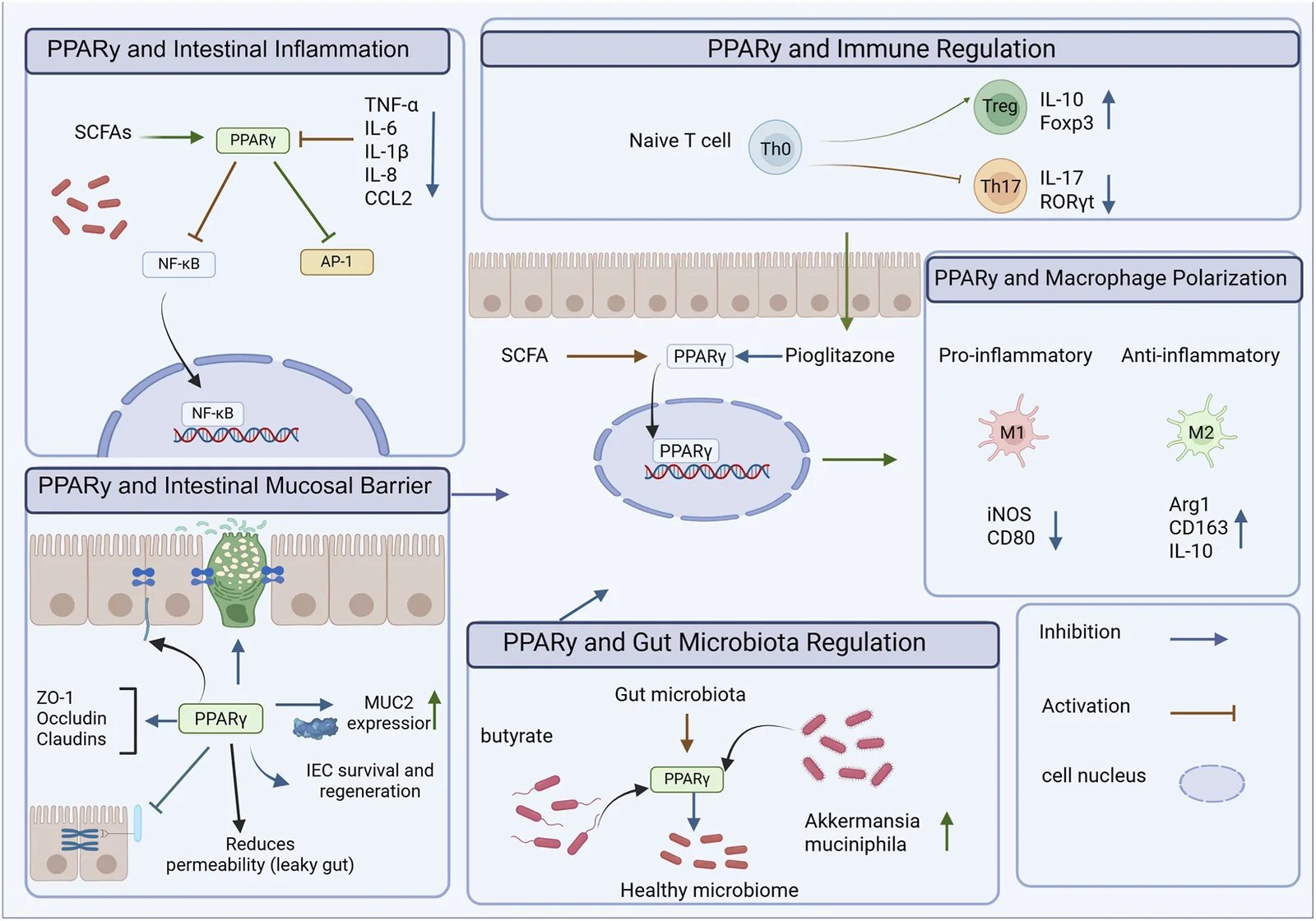
Schematic diagram illustrating the role of PPARγ in the pathogenesis of UC.

Furthermore, a dynamic regulatory network with feedback characteristics can form between PPARγ and intestinal inflammation. Inflammatory stimulation can lead to an imbalance in PPARγ signaling in the intestinal epithelium, and reduced PPARγ activity, in turn, further lifts its inhibition of pro-inflammatory pathways such as NF-κB, resulting in the sustained expression of inflammatory cytokines such as TNF-α, IL-1β, and IL-6, thereby exacerbating the local inflammatory response. It is worth noting that the anti-inflammatory effect of PPARγ is not merely manifested as the inhibition of a single inflammatory factor, but rather involves the synergistic regulation of multiple inflammatory mediators by limiting the sustained activation of inflammatory transcriptional programs (Cevallos et al., 2021). Therefore, PPARγ is best regarded as a key negative regulatory node linking inflammatory stimulation, pro-inflammatory signal transduction, and inflammatory factor expression. Activation of PPARγ in the intestinal epithelium not only alleviates colonic inflammation but also restores the epithelial metabolic homeostasis impaired under inflammatory conditions, indicating a close association between changes in PPARγ activity and the local inflammatory environment in UC (Li et al., 2021).

The above studies indicate that, during the onset and progression of UC, impaired PPARγ function may lead to a reduced ability to negatively regulate pro-inflammatory signaling, promoting the sustained activation of inflammatory pathways such as NF-κB and the release of downstream inflammatory cytokines, thereby driving the transformation of intestinal inflammation from a transient immune response to persistent pathological inflammation.

### 4.2 PPARγ and Th17/Treg immune regulation

PPARγ is widely expressed in intestinal CD4^+^ T lymphocytes and intestinal mucosal tissues and serves as a central regulatory molecule governing the adaptive immune homeostasis in UC (Martin Mena et al., 2017). An imbalance in CD4^+^ T cell subsets—particularly a disruption in the Th17/Treg cell ratio—is a key factor mediating persistent intestinal inflammatory damage. Th17 cells and Treg cells act as mutual antagonists in terms of differentiation pathways and function, jointly maintaining the dynamic balance of the intestinal immune system; a disruption of this balance induces an excessive inflammatory response, leading to colonic mucosal erosion, edema, and immune cell infiltration (Wan et al., 2020). Studies have confirmed that PPARγ expression is significantly downregulated in diseased colonic tissue from UC patients, directly disrupting the Th17/Treg immune homeostasis. This manifests as abnormal expansion of pro-inflammatory Th17 cells and suppression of protective Treg cell function, ultimately driving the progression of chronic intestinal inflammation. This suggests that abnormalities in PPARγ expression and function are important initiating factors in the immune dysregulation of UC (Xie et al., 2025).

It is worth noting that PPARγ mediates the differentiation and functional reprogramming of Th17/Treg cells by bidirectionally regulating specific transcription factors, thereby alleviating intestinal inflammatory damage in UC (Feng et al., 2025). In the pro-inflammatory pathway, activated PPARγ significantly inhibits the nuclear transcriptional activity of RORγt, a key transcription factor of Th17 cells, thereby blocking the differentiation of naive CD4^+^ T cells into Th17 cells and reducing the secretion of classic pro-inflammatory cytokines such as IL-17 and IL-22, effectively suppressing local intestinal inflammatory cascades and tissue damage (Mal et al., 2021). In the anti-inflammatory immune pathway, PPARγ specifically upregulates the expression of Foxp3, a signature transcription factor of Treg cells, promoting their proliferation, maturation, and functional activation. By secreting anti-inflammatory mediators such as TGF-β and IL-10, it enhances intestinal immune tolerance and suppresses abnormal immune responses (Cheng et al., 2021). Furthermore, PPARγ can block the inflammatory cascade by negatively regulating the NF-κB signaling pathway, while simultaneously repairing the damaged intestinal mucosal barrier and reducing antigen-mediated immune activation in the intestinal lumen (Di et al., 2024). Targeted activation of PPARγ effectively reverses the Th17/Treg ratio imbalance in a UC model and significantly improves colonic mucosal pathological damage, whereas inhibition of PPARγ activity exacerbates immune dysregulation and inflammatory infiltration.

In summary, PPARγ maintains intestinal immune homeostasis by specifically regulating the Th17/Treg immune balance, thereby providing a solid theoretical basis for molecular-targeted interventions in UC.

### 4.3 PPARγ and macrophage polarization

As a key member of the nuclear receptor superfamily, PPARγ acts as a central transcriptional regulator of macrophage phenotypic remodeling in the pathological progression of UC. In the inflammatory microenvironment of UC, intestinal lamina propria macrophages are stimulated by bacterial lipopolysaccharides and pro-inflammatory cytokines, undergo classical activation via the NF-κB and STAT1 signaling pathways, and polarize toward the M1 phenotype (Yang et al., 2022). M1-type macrophages highly express inducible nitric oxide synthase and release large amounts of TNF-α, IL-1β, and reactive oxygen species, leading to disruption of the intestinal epithelial barrier and amplification of the mucosal immune cascade (Che et al., 2025). Furthermore, upon activation by endogenous lipid ligands or exogenous agonists, PPARγ recruits the core repressor complex via protein-protein interactions, directly interfering with the binding of the NF-κB p65 subunit to DNA, thereby inhibiting the transcriptional expression of iNOS, COX-2, and various pro-inflammatory factors (Li et al., 2019). At the same time, PPARγ activation blocks STAT1 phosphorylation, thereby weakening the signaling required to maintain M1 polarization. This transcriptional-level inhibition not only reduces the concentration of local inflammatory mediators but also reduces the secretion of mature IL-1β by inhibiting the assembly and activation of the NLRP3 inflammasome, thereby disrupting the inflammatory positive feedback loop (Wu et al., 2024).

During the phase of promoting inflammation resolution and tissue repair, PPARγ, by acting in concert with transcription factors such as STAT6 and KLF4, drives macrophages to transition to the alternative, activated M2 phenotype (Xue and Wu, 2025). M2 macrophages characteristically express CD206, arginase-1, and the anti-inflammatory cytokines IL-10 and TGF-β; these effector molecules are crucial for suppressing excessive immune responses and promoting epithelial cell regeneration and extracellular matrix remodeling (Yang et al., 2022). There is a significant synergistic effect between PPARγ and IL-4/IL-13-induced STAT6 signaling; activation of PPARγ enhances STAT6 nuclear translocation and its ability to bind to M2-specific gene promoters, thereby accelerating the phenotypic shift from M1 to M2. The restoration of this polarized state is a key determinant of mucosal healing in UC. Studies have shown that in a DSS-induced colitis model, myeloid-specific PPARγ deficiency leads to a severe imbalance in the M1/M2 ratio, exacerbating colonic shortening and tissue damage, whereas treatment with a pharmacological PPARγ agonist significantly upregulates the expression of M2 markers and improves the disease activity index (Liu et al., 2022).

### 4.4 PPARγ and the gut microbiome

The relationship between PPARγ and the gut microbiota is not a unidirectional regulatory relationship; rather, it forms a bidirectional regulatory network in which “host metabolic sensing—microbiota homeostasis—mucosal barrier” are mutually coupled, playing a crucial role in the onset and progression of UC (Lavelle and Sokol, 2020). Reduced PPARγ activity can impair lipid metabolism and oxidative phosphorylation in epithelial cells and enhance iNOS expression, leading to increased local oxidative stress and nitrate levels. Increased oxygen and nitrate levels in the intestinal lumen provide a competitive metabolic advantage to facultative anaerobes, particularly Enterobacteriaceae, promoting their abnormal proliferation while suppressing the ecological niche of normal anaerobic commensal flora. This creates a vicious cycle of “diminished PPARγ function—epithelial metabolic abnormalities—increased oxygen/nitrate—proliferation of pro-inflammatory bacteria” (Caioni et al., 2021). Studies have shown that PPARγ expression in the colonic mucosa is reduced in UC, whereas activation of PPARγ can restore oxidative metabolism and the hypoxic microenvironment in colonic cells, thereby limiting the proliferation of Enterobacteriaceae. This suggests that PPARγ serves as a critical hub linking the host’s metabolic state to the structure of the gut microbiome (Fang et al., 2021). Furthermore, PPARγ is involved in maintaining the mucus barrier and epithelial integrity; it promotes mucin secretion, tight junction protein expression, and the transcription of anti-inflammatory genes, thereby limiting the migration of bacteria and microbiota-associated molecules into the mucosal lamina propria. Conversely, gut microbiota dysbiosis and its metabolites can negatively affect PPARγ activity, further compromising this protective barrier mechanism. Therefore, in UC, PPARγ dysfunction is not only the result of an imbalance in inflammatory signaling but may also be a key host factor driving the persistence of microbial dysbiosis.

On the other hand, the gut microbiota can directly or indirectly regulate PPARγ signaling through its metabolic products, forming a “microbiota-metabolite-PPARγ” regulatory axis with feedback characteristics. Short-chain fatty acids (SCFAs) produced by the normal commensal microbiota—particularly butyrate, propionate, and acetate—serve as key signaling molecules linking microbial metabolism to host cell function. Through mechanisms such as GPCRs and epigenetic regulation, they improve epithelial energy metabolism, enhance barrier function, and exert synergistic effects with PPARγ signaling (Wen et al., 2021). Previous studies have found that the abundance of butyrate-producing bacteria correlates with enhanced intestinal PPARγ signaling, suggesting that the microbial metabolic state contributes to maintaining PPARγ-dependent mucosal defense (Shon et al., 2023). Conversely, UC-associated dysbiosis is not only characterized by a reduction in SCFA-producing beneficial bacteria but may also lead to the accumulation of abnormal metabolites. Among these, the microbial-derived imidazole propionate (ImP) has been shown to inhibit PPARγ signaling, thereby further weakening the intestinal barrier and anti-inflammatory capacity, creating a positive feedback loop of “dysbiosis—increased abnormal metabolites leading to PPARγ inhibition -barrier disruption-inflammation exacerbation” (Hu et al., 2025). At the same time, PPARγ inhibition can, in turn, promote the proliferation of facultative anaerobic pro-inflammatory bacteria by altering epithelial oxygen metabolism, nitrate metabolism, and the mucosal microenvironment, thereby perpetuating the dysbiosis (Liu C. et al., 2025). Therefore, PPARγ-related interventions may be accompanied by a remodeling of the gut microbiota and metabolome, while microbiota-derived metabolites may in turn act on PPARγ and its downstream inflammatory networks, ultimately influencing NF-κB activation, oxidative stress, macrophage polarization, and epithelial barrier integrity.

### 4.5 PPARγ and the intestinal mucosal barrier

PPARγ is a key nuclear receptor involved in maintaining the homeostasis of the intestinal mucosal barrier, and its abnormal regulation is closely associated with impaired intestinal mucosal barrier integrity in patients with UC (Fang et al., 2021). The intestinal mucosal barrier is composed of the mucus layer, epithelial cells, and intercellular junctions. The mucus layer, primarily composed of MUC2, forms the first physical line of defense between the intestinal lumen and the mucosa, while tight junctions (TJs) determine the selective permeability between epithelial cells (van der Post et al., 2019). In UC, persistent mucosal damage can lead to dysfunction of goblet cells, reduced MUC2 secretion, and a decrease in mucus layer thickness, making it easier for microorganisms and their byproducts in the intestinal lumen to reach the epithelial surface. At the same time, reduced expression or abnormal localization of tight junction proteins such as ZO-1, occludin, and claudin results in loosened intercellular junctions and increased intestinal permeability (Godínez-Méndez et al., 2025). PPARγ plays a crucial role in maintaining the barrier in this process. On the one hand, PPARγ participates in the regulation of the colonic mucosal mucus barrier, promoting mucus secretion and mucin-related defensive functions, thereby enhancing the physical isolation of intestinal contents from the epithelial surface. On the other hand, PPARγ maintains the stability of tight junction complexes by influencing the expression and membrane localization of barrier-associated proteins such as ZO-1, occludin, and claudin, thereby limiting the entry of macromolecular antigens and microbial byproducts into the lamina propria via the paracellular route (Di et al., 2024). Evidence suggests that enhanced PPARγ signaling can reverse the decline in ZO-1 and occludin levels in experimental colitis and improve impaired epithelial junctional architecture; conversely, inhibition of PPARγ weakens this barrier protection (Zhou Q. et al., 2023). Therefore, under pathological conditions of UC, reduced PPARγ function may simultaneously result in “weakened mucus layer defense” and “impaired tight junction integrity,” transforming the intestinal mucosa from a highly selective physical barrier into a pathological barrier with increased permeability.

In addition, PPARγ′s regulation of the intestinal mucosal barrier is also reflected in its coordinating role in barrier damage, the remodeling of intercellular junctions, and the mucosal repair process. Under normal conditions, tight junctions are not static structures but are regulated by dynamic processes such as the cytoskeleton, endocytosis of membrane proteins, and recycling (Huang et al., 2020). Abnormal PPARγ activity can affect the stability and membrane localization of junctional proteins such as claudin and occludin, causing tight junctions to remain in a persistently disrupted state. For example, studies have shown that PPARγ is involved in regulating the membrane stability of claudin-4; when PPARγ-related signaling is impaired, endocytosis of junctional proteins increases, which can further exacerbate abnormal barrier permeability (Li et al., 2023). In UC, barrier damage can also expose the epithelial surface to increased luminal stimuli, creating a vicious cycle of “barrier disruption—mucosal exposure—persistent damage”; conversely, restoration of PPARγ helps reestablish the mucus layer and intercellular junctions, thereby reducing abnormal intestinal mucosal permeability (Zhang et al., 2025). Notably, PPARγ expression is closely associated with intestinal mucosal barrier function in UC. In models of oxidative stress-induced intestinal injury, upregulation of PPARγ improves barrier integrity, suggesting that PPARγ is not only involved in maintaining barrier structure but may also participate in the mucosal repair process following injury (Li C. et al., 2024). Therefore, in UC, low PPARγ expression or insufficient activity can lead to weakened MUC2-mediated mucus defense, disruption of tight junction structures such as ZO-1, occludin, and claudin, and increased intestinal permeability. Ultimately, this causes the intestinal mucosal barrier to shift from a steady-state condition of “structural integrity and selective permeability” to a pathological state characterized by “mucus deficiency, loose junctions, and abnormal leakage.”

## 5 Mechanisms of action of natural compounds in modulating PPARγ-Related signaling pathways to intervene in UC

### 5.1 PPARγ/NF-κB signaling pathway

NF-κB is a key transcription factor linking innate immune recognition to the transcription of inflammatory genes; the classical pathway is primarily mediated by the p65/p50 heterodimer (Florio et al., 2022). Under resting conditions, NF-κB binds to IκBα and remains in the cytoplasm; upon stimulation by pathogen-associated molecules and inflammatory mediators, the IKK complex is activated and promotes the phosphorylation and degradation of IκBα, leading to the release of p65 and its nuclear translocation, which in turn initiates the expression of inflammation-related genes (Li Y. et al., 2024). In UC, the intestinal mucosa is continuously stimulated by microbial products and injury signals, which can lead to abnormal activation of the TLR4/NF-κB pathway, promoting the expression of TNF-α, IL-1β, IL-6, and iNOS, and driving pro-inflammatory polarization of macrophages, ROS accumulation, and inflammatory cell infiltration, thereby forming a persistent inflammatory amplification loop; simultaneously, excessively activated NF-κB can exacerbate epithelial damage, reduce the expression of tight junction proteins such as ZO-1 and Occludin, and further disrupt the homeostasis of the mucosal barrier (Jia et al., 2026). As a key negative regulator of NF-κB, PPARγ inhibits IκBα phosphorylation and p65 nuclear translocation, thereby limiting the production of pro-inflammatory mediators at the transcriptional level and improving the inflammatory phenotype of macrophages. Therefore, an imbalance in PPARγ/NF-κB regulation represents a critical molecular link between the persistent activation of the immune-inflammatory response in UC and mucosal barrier damage; inhibiting abnormal NF-κB activation helps block the inflammatory cascade and promote the restoration of intestinal mucosal homeostasis (Table 1; Figures 3–5).

**TABLE 1:** Natural compounds modulate PPARγ-Related signaling pathways to intervene in UC.

Extract	*Origination*	Structure	Dosage	Model	Biological effects	Results	Limitations	References
Luteolin	*Lonicera japonica* Thunb	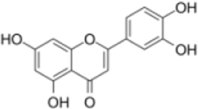	12.5,25,50 mg/kg	UC model	DAI↓p-MAPK↑PPARγ↑iNOS↓TNF-α↓Arg1↑CD206↑	Improve shortened colon length while restoring the integrity of the intestinal mucosal barrier	Reliance on acute DSS models and single cell lines fails to adequately simulate the heterogeneity and chronic relapsing nature of UC.	Yang et al. (2025)
			6.25,12.5.25 μM	RAW264.7 cells				
Madecassic acid	*Centella asiatica* (L.) Urb	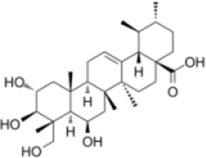	12.5,25 mg/kg	UC model	p-MAPK↑PPARγ↑ACC1↓	Significantly reduces disease activity indices, alleviates colonic shortening and myeloperoxidase activity, and repairs pathological damage to the intestinal mucosa	Limited to a single chemically induced model and short-term intervention periods, its applicability in chronic, relapsing colitis or microbiome regulation has not been fully validated	Xu et al. (2017)
Gleditsia sinensis thorn extract	*Gleditsia sinensis* Lam	---	80,160,320 mg/kg	UC model	TNF-α↓IL-6↓IL-1β↓IL-10↑PPARγ/AMPK↑	Effectively alleviated symptoms of DSS-induced colitis in mice and reduced the disease activity index and colon shortening	The lack of evaluation of its *in vivo* pharmacokinetic characteristics and distribution in colonic tissue limits the availability of robust evidence to support its translation into clinical practice	Liu et al. (2025a)
Emodin	*Rheum* spp.	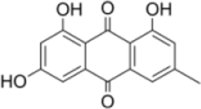	5,10,20 mg/kg	UC model	PPARγ↑iNOS↓NF-κB p65↓	Reduces nitrate levels in the intestinal mucosa and regulates oxidative phosphorylation and citrulline metabolism	*In vivo* validation of rescue effects has not been conducted using PPARγ knockout animals	Luo et al. (2022)
			5,10,20 μM	Caco-2 cells				
Paeonol	*Paeonia suffruticosa* Andr	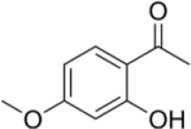	200,400 mg/kg	UC model	TNF-α↓IL-6↓ROS↓PPARγ↑	Reduces inflammatory infiltration and apoptosis in colonic tissue and maintains the integrity of the intestinal epithelial barrier	The pharmacokinetic characteristics and long-term safety of paeonol have not been studied, and differences in efficacy among different UC subtypes also require further clarification	Cheng et al. (2024)
			20,40,80 μM	RAW264.7 cells				
Ginsenoside Rb1	*Panax ginseng* C.A.Mey	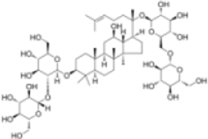	20,40,80 mg/kg	UC model	TNF-α↓IL-6↓IL-10↑ZO-1↑Occludin↑E-cadherin↑	Suppress inflammation and restore gut homeostasis	Functional validation based on correlation analysis of protein expression, without target-specific interventions	Zhou et al. (2024)
18β-GA	*Glycyrrhiza uralensis* Fisch	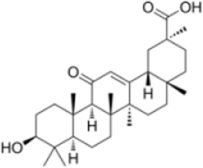	40 mg/kg	UC model	p-NF-κB↓PPAR-γ↑	Significantly alleviated colon shortening, elevated DAI index, and histopathological damage	A direct causal relationship between PPAR-γ and NF-κB has not been verified through genetic intervention, and there is a lack of pharmacokinetic and safety evaluations	Zhu et al. (2025)
			62.5 μM	MCECs cells				
Honokiol	*Magnolia officinalis* Rehder & E.H.Wilson	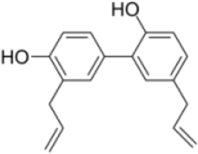	2.5.5 mg/kg	UC model	TNF-α↓IL-6↓IL-1β↓IFN-γ↓	Inhibits intestinal inflammatory responses	The causal relationship of the pathway has not been confirmed in mice with conditional PPAR-γ knockout or NF-κB reporter genes	Wang et al. (2022)
			5.10 μM	RAW264.7 cells				
Aesculin	*Fraxinus chinensis* Roxb	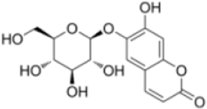	1.5 mg/kg	UC model	NF-κB↓PPAR-γ↑iNOS↓TNF-α↓IL-1β↓	Improves weight loss, colon shortening, and mucosal erosion in mice with colitis	Regulation of the Th1/Th2 and Treg/Th17 immune balance in the gut has not been investigated	Tian et al. (2019)
			300,400,500 μM	RAW264.7 cells				
D-pinitol	*Ceratonia siliqua*	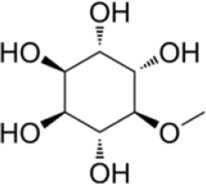	10,20,40 mg/kg	UC model	HO-1↑NQO1↑	Alleviates colon shortening, tissue damage, and inflammatory cell infiltration	Based solely on a single acute DSS model; not validated in chronic or T-cell-mediated colitis	Lin et al. (2021)
Magnolol	*Magnolia officinalis* Rehder & E.H.Wilson	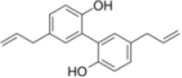	25,50,100 mg/kg	UC model	TNF-α↓IL-1β↓IL-12↓NF-κB↓PPAR-γ↑	Improved weight loss, elevated disease activity index, colon shortening, and pathological damage to colon tissue in model mice	Lack of *in vitro* mechanisms supporting the presence of colonic epithelial cells, as well as data on long-term safety and *in vivo* pharmacokinetics	Shen et al. (2018)
Acalypha australis L	Euphorbiaceae	---	125,250,500 mg/kg	UC model	IL-6↓IL-1β↓TNF-α↓MUC2↑Occludin↑ZO-1↑	Alleviates pathological damage to the colon, such as inflammatory infiltration and destruction of crypts	Limited to an *in vivo* mouse model of chronic colitis; validation of *in vitro* pathway blockade in intestinal epithelial cells is lacking	Quan et al. (2025)
Geniposide	*Gardenia jasminoides* J.Ellis	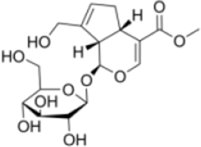	50,100,200 mg/kg	UC model	IL-6↓IL-1β↓TNF-α↓Occludin↑ZO-1↑	Reduce Neutrophil infiltration and pathological damage to the intestinal mucosa	Relying solely on chemically induced models of isolated colitis makes it difficult to replicate the heterogeneous clinical phenotype of ulcerative colitis	Zhang et al. (2017)
			50,100,200 μg/mL	Caco-2 cells				
Purslane extract	*Portulaca oleracea* L	---	100 mg/kg	UC model	IL-6↓IL-1β↓TNF-α↓MPO↓	Alleviates inflammatory infiltration, crypt defects, and tissue erosion in the colonic mucosa	It is difficult to replicate clinical inflammatory bowel disease using only a single animal model of acute colitis	Kong et al. (2018)
Dioscin	*Dioscorea nipponica* Makino	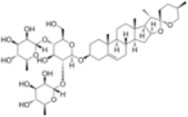	40,80,160 mg/kg	UC model	IL-1β↓TNF-α↓	Reduces pro-inflammatory mediators, increases the expression of anti-inflammatory factors, and repairs pathological damage to the intestinal mucosa	Only assesses the expression levels of key proteins in the pathway, enzymes involved in carbohydrate and lipid metabolism, and markers of macrophage polarization	Wu et al. (2021)
			0.625,1.25,2.5 μM	RAW264.7 cells				
Berberine	*Coptis chinensis* Franch	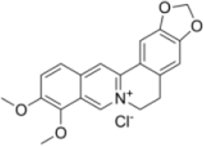	50,100 mg/kg	UC model	IL-6↓IL-1β↓TNF-α↓PPAR-γ↑	Significantly improved pathological manifestations of colitis in model mice, such as weight loss and colon shortening, and alleviated intestinal mucosal damage	Due to the lack of rigorous validation using PPAR-γ knockout mice, the direct molecular interaction mechanisms by which PPAR-γ regulates mTOR/HIF-1α remain unclear	He et al. (2026)
			0,3,6,12,24,48 μM	RAW264.7 cells				
Crassula tetragona L	---	---	200,400 mg/kg	UC model	TNF-α↓IL-6↓IL-10↑SOD↑GSH↑MDA↓	Inhibiting intestinal inflammation and oxidative imbalance	It is not possible to clearly identify direct interactions between phenolic bioactive molecules and PPARγ/SIRT1 proteins, and there are significant limitations in elucidating the mechanisms of action at the molecular target level	Raslan et al. (2026)
Cinobufotalin	*Bufo gargarizans* Cantor	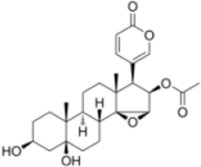	0.5.2 mg/kg	UC model	IL-6↓TNF-α↓IKK/IκBα/p65↓JNK/p38/ERK↓	Reduces inflammatory cell infiltration in the colonic mucosa and repairs damage to the intestinal barrier	A germ-free mouse model lacking gut microbiota was not established to conduct microbiota-specific targeted intervention experiments	Feng et al. (2025)
Lentinus edodes β-glucan	*Lentinula edodes*	---	500,1000 mg/kg	UC model	TNF-α↓IL-1β↓IL-6↓iNOS↓MPO↓MDA↓NO↓	Alleviating oxidative stress and intestinal inflammation	Does not involve pathological subtypes such as chronic inflammation or recurrent enteritis	Shi et al. (2016)
			100,200,400 μg/mL	RAW264.7 cells				
Licorice flavonoid extract	*Glycyrrhiza uralensis* Fisch	---	100 mg/kg	UC model	MAPK↓NF-κB p65↓TNF-α↓IL-6↓	Improves conditions such as colonic shortening and mucosal ulcers	Key gut metabolites, such as short-chain fatty acids, were not measured; therefore, the downstream metabolic mechanisms by which the microbiota regulates anti-inflammatory responses have not been fully elucidated	Zhu et al. (2026)
Indole-3-propionic acid	---	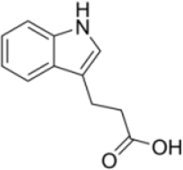	20 mg/kg	UC model	HK2↓ACSL1↑CPT1A↑	Reduces the colitis disease activity index and alleviates pathological damage to colonic tissue	No *in vivo* pharmacokinetic, tissue distribution, or long-term toxicological safety evaluations of IPA have been conducted	Li et al. (2025)
			10,100 μmol/L	RAW264.7 cells				
Naringin	*Citrus maxima (Burm.)* Merr	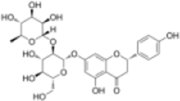	25,50,100 mg/kg	UC model	TNF-α↓IL-1β↓IL-6↓NLRP3↓PPAR-γ↑	Improvement in the UC mouse disease activity index, colonic shortening, and intestinal histopathological lesions	There are no human clinical trials to support the potential for translating drug efficacy, and the upstream molecular targets that regulate PPARγ have not been identified	Cao et al. (2018)
			20 μM	RAW264.7 cells				
Moringin	*Moringa oleifera* Lam	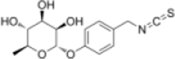	5.15 mg/kg	UC model	TNF-α↓IL-1β↓MDA↓MPO↓	Alleviated weight loss, colon shortening, bloody stools, and histopathological lesions in UC mice	Unutilized patient colon organoids do not replicate the actual pathological environment of the human intestine, and no *in vivo* pharmacokinetic or repeated-dose toxicity evaluations have been conducted	Zhang et al. (2024)
			5,10,20 μM	Caco-2 cells				
Paris polyphylla ethanol extract	*Paris polyphylla* Sm	---	50,100 mg/kg	UC model	TNF-α↓IL-1β↓IL-6↓IL-10↑	Restore the structure of the intestinal mucosa and immune balance	The study was limited to a chemically induced mouse colitis model; no active components were isolated from Paris polyphylla to verify direct binding to specific targets	He et al. (2023)
Alliin	*Allium sativum* L	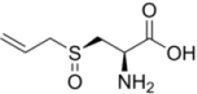	12.5,25,50 mg/kg	UC model	TNF-α↓IL-6↓iNOS↓NO↓MPO↓MDA↓	Alleviates inflammatory cell infiltration in colonic tissue, crypt destruction, shortening of the intestinal tract, and splenomegaly	No comparisons were made regarding dosage gradients, and no efficacy analyses were conducted for different intervention durations or routes of administration; gaps in understanding the upstream and downstream regulatory interactions within the molecular pathway remain	Shi et al. (2017)
			6.25,12.5.25 μM	RAW264.7 cells				

**FIGURE 3:**
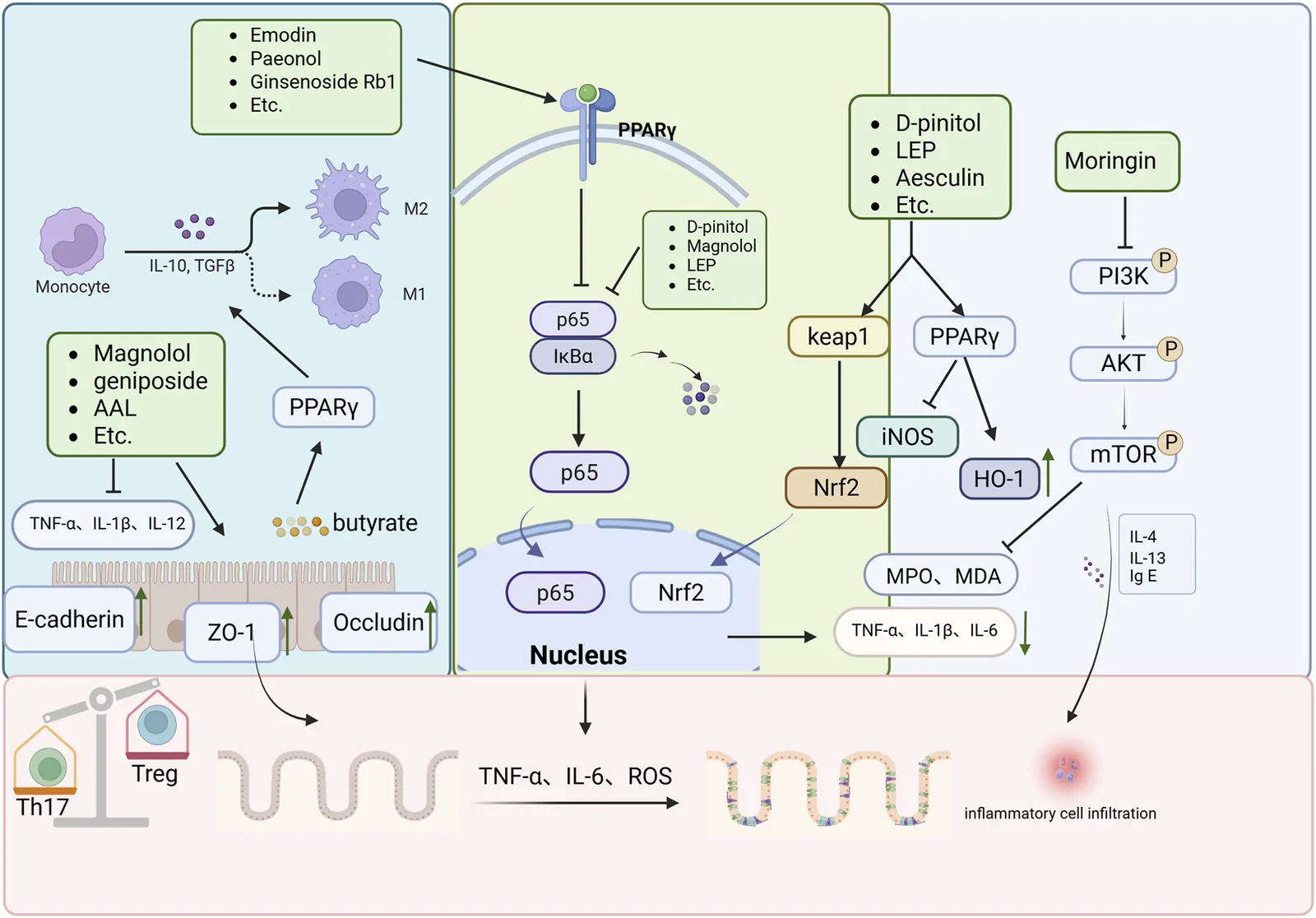
Schematic diagram illustrating the mechanism by which natural compounds modulate the PPARγ/NF-κB signaling pathway to treat UC.

**FIGURE 4:**
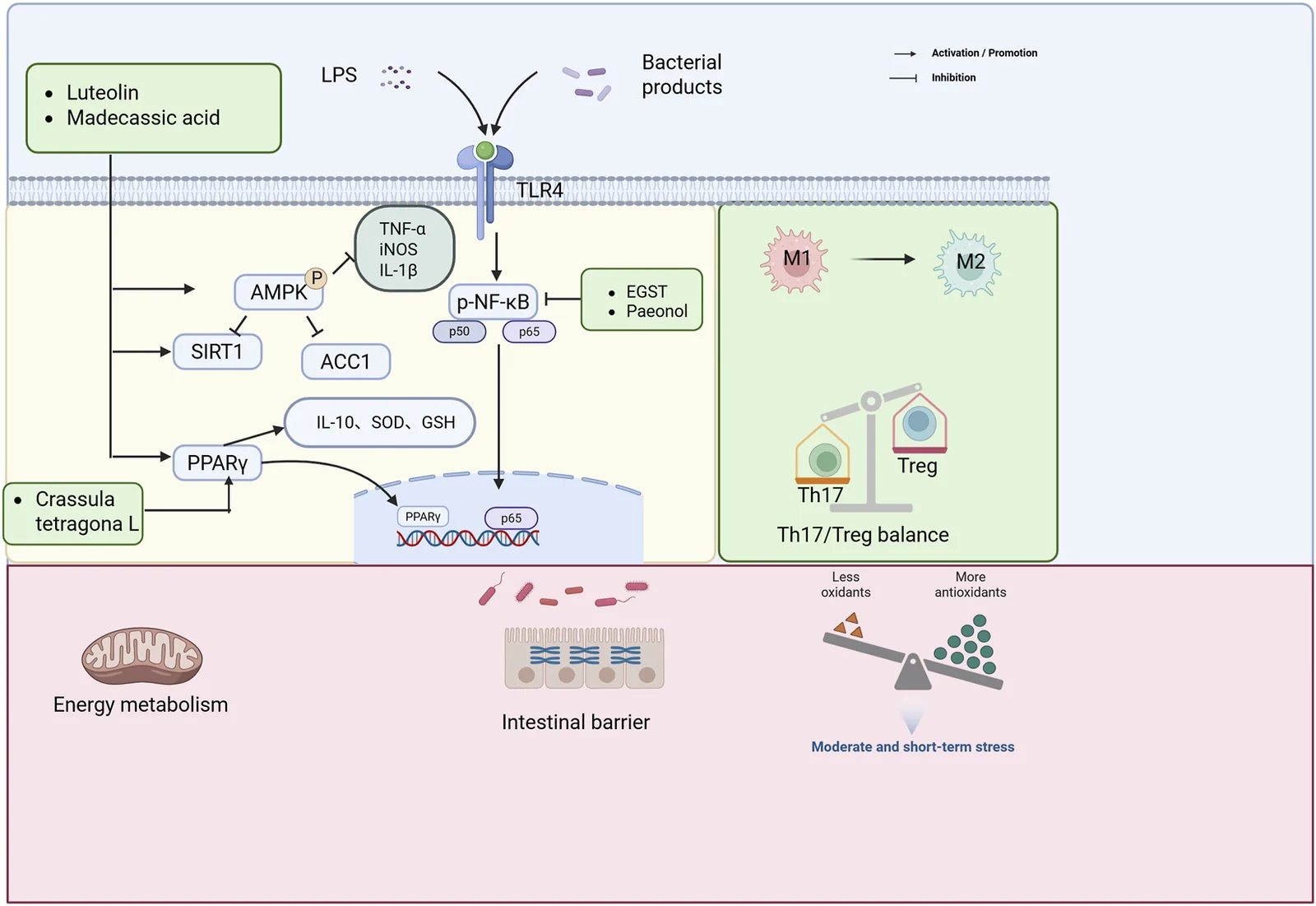
Schematic diagram illustrating the mechanism by which natural compounds modulate the PPARγ/AMPK/SIRT1 signaling pathway to treat UC.

**FIGURE 5:**
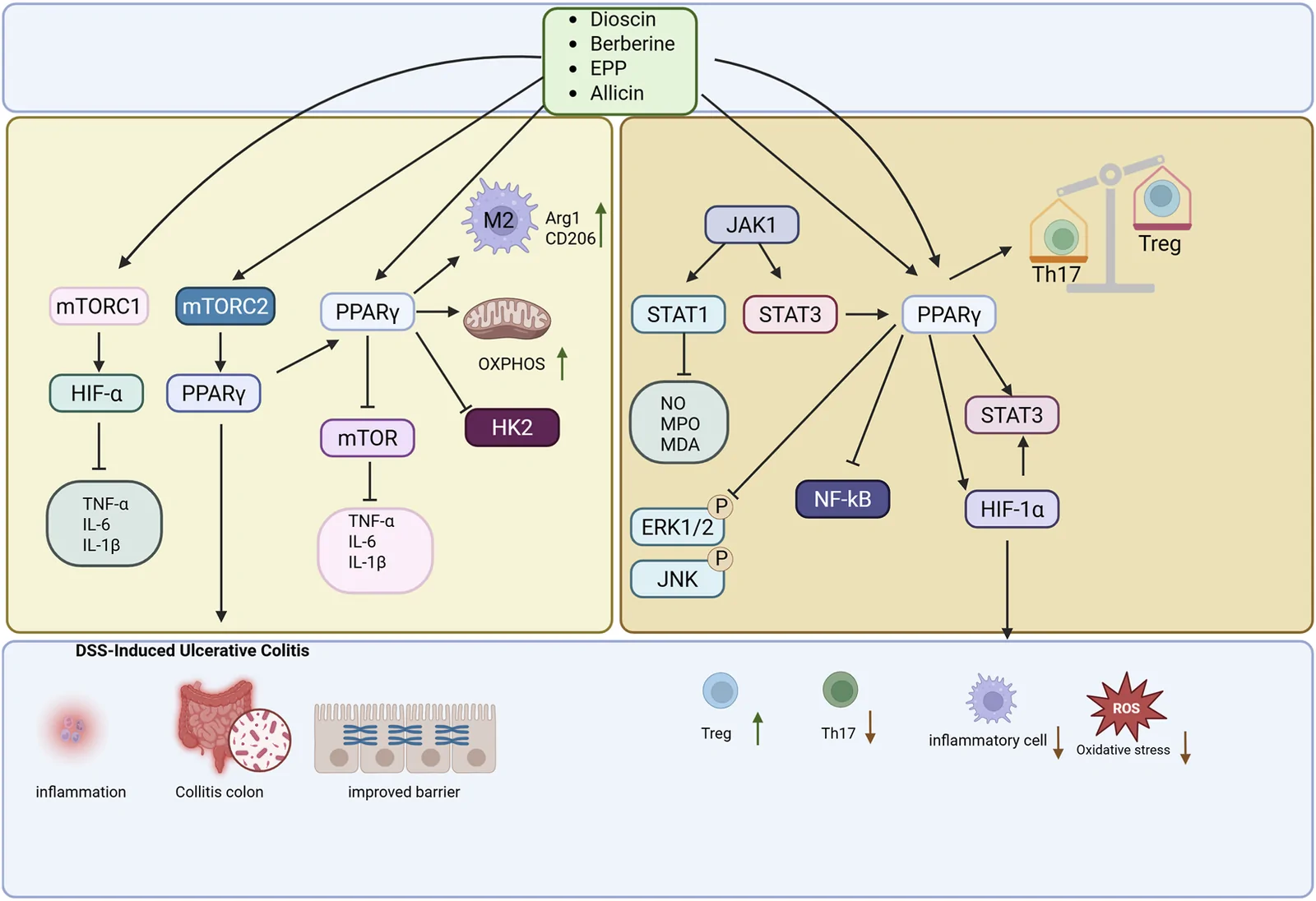
Schematic diagram illustrating the mechanism by which natural compounds modulate the PPARγ/mTOR/STAT signaling pathway to treat UC.

Emodin is a natural anthraquinone compound found in *Rheum palmatum* L. (Polygonaceae) and *Reynoutria japonica* Houtt. (Polygonaceae), possessing anti-inflammatory, antioxidant, and intestinal mucosal protective effects. Luo et al. evaluated emodin (5, 10,20 mg/kg/day) in a 10-day study using a mouse model of UC, in combination with mesalazine, rosiglitazone, and the PPARγ antagonist GW9662. Emodin alleviated colonic inflammatory damage in a dose-dependent manner. *In vitro* experiments showed that emodin (5,10,20 μM) enhanced PPARγ-related signaling and inhibited iNOS/NF-κB p65 signaling, while PPARγ knockdown attenuated these effects. The study further indicated that emodin treatment improves epithelial cell hypoxia, oxidative phosphorylation, citrulline metabolism, and gut microbiota homeostasis (Luo et al., 2022). The combination of pharmacological experiments and PPARγ knockdown experiments provides relatively strong evidence that PPARγ plays a functional role in the protective effects of emodin. However, since the direct binding of the compound to PPARγ and receptor activation were not evaluated, the current evidence only supports a PPARγ-dependent mechanism rather than establishing PPARγ as a direct molecular target of emodin.

Paeonol is a naturally occurring phenolic compound found in *Paeonia × suffruticosa* Andrews (Paeoniaceae) and exhibits anti-inflammatory, antioxidant, and immunomodulatory properties. Cheng et al. studied Paeonol using LPS-stimulated RAW264.7 macrophages and NCM460 cells in combination with a DSS-induced UC mouse model. *In vitro* experiments showed that Paeonol (20,40,80 μM) upregulated PPARγ expression, inhibited NF-κB p65 activation, reduced IL-6, TNF-α, and ROS levels, and promoted the formation of the M2-like macrophage phenotype; pharmacological blockade with GW9662 (10 μM) attenuated these effects. In in vivo experiments, paeonol (200,400 mg/kg/day for 10 consecutive days) alleviated DSS-induced disease activity, colonic damage, and inflammatory cytokine production, with SASP (200 mg/kg/day) serving as the positive control (Cheng et al., 2024). These findings support the functional involvement of PPARγ-related signaling pathways in the anti-inflammatory effects of paeonol. However, pharmacological antagonism based on GW9662 and changes in PPARγ expression alone are insufficient to prove direct receptor activation or target binding; gene disruption experiments, as well as direct binding assays or assessments of PPARγ transcriptional activity, are required before paeonol can be considered a compound that acts directly on PPARγ.

Ginsenoside Rb1 (GRb1) is a major ginsenoside found in *Panax ginseng* C.A.Mey. (Araliaceae), exhibiting anti-inflammatory, immunomodulatory, and barrier-protective effects. Zhou et al. evaluated GRb1 using a 3% DSS-induced murine model of UC, in which mice were administered GRb1 (20,40,80 mg/kg/day) via oral gavage for 10 consecutive days. GRb1 improved DAI scores, colonic shortening, and histopathological damage in a dose-dependent manner; it reduced TNF-α and IL-6 levels, increased IL-10 levels, and restored the expression of tight junction-associated proteins ZO-1, occludin, and E-cadherin (Zhou et al., 2024). Although these changes were accompanied by the regulation of PPARγ-related signaling pathways, the study did not employ pharmacological blockade, gene disruption, or complementary cellular models of PPARγ/VDR. Therefore, while the available evidence establishes an association between GRb1 treatment and PPARγ-related molecular changes, it is insufficient to demonstrate that PPARγ is functionally essential for GRb1’s action or is directly targeted by GRb1.

18β-Glycyrrhetinic acid (18β-GA) is a triterpenoid associated with the constituents of *Glycyrrhiza uralensis* Fisch. ex DC. (Fabaceae), exhibiting anti-inflammatory, antioxidant, and immunomodulatory effects. Zhu et al. evaluated its effects using a DSS-induced murine model of UC and a macrophage-intestinal epithelial cell co-culture system. Following induction with 4% DSS, mice received 18β-GA (40 mg/kg/day) for 12 consecutive days, with 5-ASA (40 mg/kg/day) serving as the positive control. Treatment improved DAI scores, colonic damage, and tissue inflammation, accompanied by changes in the composition of the gut microbiota. In the macrophage-MCEC co-culture system, 18β-GA (62.5 μM) promoted epithelial cell migration and mucosal repair. These effects were accompanied by enhanced PPARγ-related signaling pathways and reduced levels of NF-κB p65 phosphorylation (Zhu et al., 2025). However, since neither PPARγ gene disruption experiments nor direct target binding assays were conducted, these data only support an association between 18β-GA treatment and the PPARγ/NF-κB axis, rather than proving that it directly activates PPARγ. Furthermore, it remains inconclusive whether the observed changes in the microbiota have a causal relationship with the therapeutic effects.

Honokiol (HKL) is a naturally occurring biphenolic compound found in *Magnolia officinalis* Rehder & E.H.Wilson (Magnoliaceae), possessing anti-inflammatory, antioxidant, and mucosal protective effects. Wang et al. studied HKL using a DSS-induced murine model of ulcerative colitis and LPS-stimulated RAW264.7 macrophages. Mice were treated with HKL (2.5 or 5 mg/kg/day) for five consecutive days, with mesalazine (66.7 mg/kg/day) serving as a positive control. The results showed improvements in DAI scores, colonic shortening, histopathological damage, and inflammatory mediator expression. In LPS-stimulated RAW264.7 cells, HKL (5 and 10 μM) enhanced PPARγ-related signaling and inhibited TLR4/NF-κB signaling and GSDMD-associated apoptosis, whereas GW9662 attenuated these protective effects (Wang et al., 2022). Therefore, these pharmacological antagonistic effects support a functional role for PPARγ in the cellular effects of HKL. However, due to the lack of gene disruption and direct target-binding experiments, it is not possible to conclude that HKL directly binds to or specifically activates PPARγ.

Aesculin is a coumarin-derived bioactive compound from the bark of *Fraxinus chinensis* Roxb. (Oleaceae) and its congeneric species, and exhibits anti-inflammatory and immunomodulatory effects. Tian et al. investigated Aesculin using a mouse model of DSS-induced UC and LPS-stimulated RAW264.7 macrophages. BALB/c mice were administered Aesculin (1.5 mg/kg) after exposure to 2.5%DSS, with 5-ASA (50 mg/kg) serving as a positive control. Aesculin improved DAI scores, reduced colonic shortening, and alleviated histopathological damage, while also decreasing the expression of iNOS, IL-1β, and TNF-α. In RAW264.7 cells, relatively high concentrations of Aesculin (300–500 μM) inhibited NF-κB p65 activation and promoted PPARγ nuclear translocation, while GW9662 partially attenuated its anti-inflammatory effects (Tian et al., 2019). These pharmacological data support the involvement of PPARγ-related signaling pathways but do not establish the direct involvement of PPARγ. Furthermore, the relatively high concentrations used in in vitro experiments raise questions regarding pharmacological relevance and potential nonspecific effects. Therefore, direct binding assays, receptor functional assays, gene disruption experiments, and dose-response studies are needed before a specific PPARγ-mediated mechanism can be established.

D-Pinitol is a naturally occurring cyclitol found in several plant species, including *Glycine* max (L.) Merr. (Fabaceae), *Ceratonia siliqua* L. (Fabaceae), and *Bruguiera gymnorhiza* (L.) Lam. (Rhizophoraceae), and possesses antioxidant and anti-inflammatory properties. Lin et al. evaluated the effects of D-pinol in a 3% DSS-induced mouse model of UC. Oral administration of D-pinol (10,20,40 mg/kg/day for seven consecutive days), with SASP (200 mg/kg/day) as a positive control, alleviated weight loss, DAI scores, colonic shortening, histopathological damage, and the production of inflammatory cytokines. Western blot analysis revealed increased PPARγ expression, accompanied by reduced phosphorylation levels of NF-κB p65/IκBα and enhanced Nrf2/ARE-related signaling. Molecular docking predicted potential interactions between D-pinol and PPARγ residues, including Ser342 and Arg288 (Lin et al., 2021). It should be noted that molecular docking represents only a computational hypothesis and does not constitute experimental evidence of physical binding. Since no intracellular PPARγ disruption experiments, receptor function assays, or direct binding assays have been conducted, the current evidence is insufficient to confirm that the effects of D-pinolol are PPARγ-dependent.

Magnolol is a naturally occurring biphenolic compound found in *M. officinalis* Rehder & E.H.Wilson (Magnoliaceae), possessing anti-inflammatory and mucosal protective effects. Shen et al. evaluated magnolol using a 2.5% DSS-induced ulcerative colitis (UC) model in C57BL/6 mice and an MLN cell inflammation model. Magnolol (25, 50, or 100 mg/kg/day for five consecutive days) improved DAI scores, colon shortening, and histopathological damage; reduced levels of TNF-α, IL-1β, and IL-12; and restored the expression of ZO-1 and occludin. These effects were accompanied by increased PPARγ expression and reduced phosphorylation levels of NF-κB p65/IκBα (Shen et al., 2018). However, this study did not include a positive drug control group, nor did it conduct experiments to verify whether PPARγ is functionally essential for these effects. Therefore, although the current findings suggest an association between magnolol treatment and PPARγ/NF-κB-related molecular changes, they do not establish its PPARγ dependence or direct targeting of PPARγ.

An extract of *Acalypha australis* L. (Euphorbiaceae; AAL) has been reported to possess anti-inflammatory and intestinal barrier-protective effects. Quan et al. evaluated AAL (125, 250, or 500 mg/kg/day for 45 consecutive days) in a DSS-induced chronic colitis model, using 5-ASA (100 mg/kg/day) as a positive control. AAL improved weight loss, DAI scores, colonic shortening, and histopathological damage; reduced levels of IL-6, IL-1β, and TNF-α; and restored the expression of MUC2, ZO-1, and occludin. UPLC-QTOF-MS identified its major components as including begaptole and corilagin. Proteomics and GSEA analyses indicated that FABP4/PPARγ/NF-κB-related signaling pathways were involved, while GW9662 attenuated the protective effects of AAL (Quan et al., 2025). These findings support the functional contribution of PPARγ-related signaling pathways to the activity of this extract. However, since AAL is a mixture with a complex chemical structure and its active components have not yet been functionally identified, it is currently impossible to confirm that it directly activates PPARγ or to attribute this effect to a specific compound.

Geniposide (GE) is an iridoid glycoside found in *Gardenia jasminoides* J. Ellis (Rubiaceae), possessing anti-inflammatory and antioxidant effects. Zhang et al. evaluated the effects of GE using a mouse model of DSS-induced UC and LPS-stimulated Caco-2 intestinal epithelial cells. GE (50, 100, or 200 mg/kg/day) improved DAI scores, reduced colonic shortening, and alleviated histopathological damage, while lowering levels of TNF-α, IL-6, and IL-1β and restoring the expression of ZO-1 and occludin. Similar anti-inflammatory and barrier-protective changes were also observed in Caco-2 cells treated with GE (50–200 μg/mL). These effects were accompanied by increased PPARγ expression and reduced levels of NF-κB p65/IκBα phosphorylation (Zhang et al., 2017). However, the study did not involve pharmacological or genetic intervention targeting PPARγ. Therefore, while these findings support an association between GE treatment and PPARγ/NF-κB-related signaling, they are insufficient to demonstrate that these effects depend on PPARγ, receptor activation, or direct target binding.

Extract of *Paris polyphylla* Sm. (Melanthiaceae) (EPP) is the plant extract derived from the rhizome of *P. polyphylla* Sm. (Melanthiaceae). and possesses pharmacological effects such as anti-inflammatory properties. Kong et al. evaluated this extract (100 mg/kg) in a 3% DSS-induced acute UC model, using mesalazine (7.4 mg/kg) as a positive control. Following treatment, DAI scores decreased, colon length recovered, MPO activity and histopathological inflammation were reduced, and the expression of TNF-α, IL-6, and IL-1β decreased. These effects were accompanied by the restoration of DSS-induced PPARγ downregulation, inhibition of the NF-κB signaling pathway, and regulation of Bcl-2/Bax/caspase-3-mediated apoptosis (Kong et al., 2018). However, the restoration of PPARγ expression alone does not prove that PPARγ mediates the therapeutic effects. In the absence of pharmacological blockade, gene disruption, or direct target-binding experiments, PPARγ should therefore be considered a relevant signaling component rather than an established molecular target of the extract.

### 5.2 PPARγ/MAPK signaling pathway

MAPK is a critical serine/threonine protein kinase cascade system through which cells sense and transmit signals related to inflammation, oxidative stress, and immune stimulation. It primarily comprises branches such as ERK1/2, JNK, and p38 MAPK, which regulate the activity of transcription factors and the expression of inflammation-related genes through stepwise phosphorylation. During the development of UC, luminal antigens, dysbiosis, and oxidative stress can continuously activate the MAPK signaling pathway, promoting the phosphorylation of JNK, p38, and ERK. This interacts synergistically with inflammatory transcriptional networks such as NF-κB and AP-1 to induce the expression of inflammatory mediators including TNF-α, IL-1β, IL-6, and iNOS, thereby further exacerbating mucosal inflammatory infiltration and barrier damage (Li et al., 2025a). At the same time, MAPK can also participate in intestinal immune and metabolic homeostasis by regulating PPARγ activity; its abnormal activation can affect macrophage glucose and lipid metabolism as well as M1/M2 phenotypic switching, thereby maintaining a pro-inflammatory microenvironment. Studies have shown that inhibiting JNK/MAPK activity reduces HK2-mediated glycolysis and limits the pro-inflammatory function of M1 macrophages, whereas PPARγ activation promotes fatty acid oxidation and M2 phenotype formation. Furthermore, a functional link exists between MAPK and PPARγ at the phosphorylation level; the activation of MAPKs such as ERK and JNK can influence the phosphorylation of PPARγ at Ser112 and its transcriptional activity; Blocking the phosphorylation of JNK, ERK1/2, and p38 reduces NF-κB/AP-1 activity and the release of pro-inflammatory mediators, thereby alleviating colonic mucosal damage (Shi et al., 2016). Therefore, abnormal MAPK activation collectively drives the persistence of UC inflammation through inflammatory transcription, immunometabolism, and PPARγ-related regulation, serving as a critical signaling hub linking intestinal inflammation to immune homeostasis imbalance.

Cinobufotalin (CB) is a naturally occurring bufadienolide found in the skin and venom of the toad *Bufo gargarizans* Cantor (Bufonidae), and exhibits anti-inflammatory and immunomodulatory activities. Feng et al. evaluated the effects of umbilical CB in a 3% DSS-induced UC model in BALB/c mice. The treatment regimen consisted of consecutive 6-day administrations of CB (0.5.2 mg/kg), with mesalazine (100 mg/kg) serving as the positive control. CB attenuated DAI scores, colonic shortening, and histopathological damage; reduced the expression of inflammatory cytokines; improved intestinal barrier function; and modulated the composition of the gut microbiota and the Th17/Treg balance. The study further demonstrated the inhibitory effects on the JNK/p38/ERK MAPK and NF-κB signaling pathways. In parallel experiments, butyrate-treated CD4^+^ T cells exhibited changes in the PPARγ/CD36 axis and Th17/Treg-related markers, whereas the PPARγ antagonist T0070907 attenuated the protective effects observed *in vivo* (Feng et al., 2025). These findings support the functional involvement of PPARγ-related signaling in the overall therapeutic response; however, they do not prove that CB acts directly on PPARγ, particularly since no experiments were conducted to assess direct interactions between CB and PPARγ or to determine CB-specific cellular targets.

Lentinula edodes β-glucans (LEBG) are naturally occurring polysaccharides derived from *Lentinula edodes* (Berk.) Pegler (Omphalotaceae) and possess anti-inflammatory and immunomodulatory properties. Shi et al. evaluated the effects of LEBG in an ICR mouse model of DSS-induced UC and in an LPS-stimulated RAW264.7 macrophage model. LEBG alleviated colonic damage and reduced the levels of MPO, MDA, NO, TNF-α, IL-1β, IL-6, and iNOS. At the molecular level, LEBG inhibited JNK, ERK1/2, and p38 MAPK signaling, reduced inhibitory phosphorylation of PPARγ at Ser112, and was accompanied by enhanced PPARγ activity; MAPK inhibitors further confirmed the regulatory link between MAPK signaling and PPARγ(Shi et al., 2016). These data provide evidence that LEBG exerts its effects by modulating the MAPK–PPARγ regulatory network rather than by directly acting on PPARγ. Since no PPARγ-specific pharmacological or genetic interference experiments were conducted, nor were any direct target binding assays performed, it remains unclear whether PPARγ plays a functional role in the protective effects of LEBG.

Licorice flavonoid extract (LF), derived from the roots of *G. uralensis* Fisch. ex DC. (Fabaceae), contains various flavonoid constituents and exhibits anti-inflammatory and immunomodulatory effects. Zhu et al. used a 3% DSS-induced model of UC in C57BL/6 mice to evaluate the efficacy of LF (100 mg/kg/day for 15 consecutive days), with berberine (100 mg/kg/day) serving as the positive control. LF improved DAI scores, colonic shortening, and histopathological damage; reduced TNF-α and IL-6 levels; restored the expression of ZO-1, occludin, and tight junction protein-1; and regulated gut microbiota dysbiosis (Zhu et al., 2026). These effects were accompanied by increased PPARγ expression and reduced phosphorylation levels of p38 MAPK and NF-κB p65. However, since the study relied primarily on changes in tissue-level expression and did not involve PPARγ-specific pharmacological or genetic interference, the data only suggest an association with the PPARγ/MAPK/NF-κB signaling pathway rather than PPARγ dependence. Furthermore, given that LF is a multicomponent extract, the specific components responsible for the observed molecular effects and their precise targets remain unclear.

Indole-3-propionic acid (IPA) is an indole metabolite produced by the gut microbiota through the metabolism of tryptophan; it possesses anti-inflammatory and immunomodulatory effects. Li et al. evaluated IPA using a 3% DSS-induced UC model in C57BL/6J mice, as well as LPS-induced M1-type and IL-4-induced M2-type polarization models established using RAW264.7 macrophages. IPA improved DAI scores and histopathological damage and shifted macrophage polarization from the M1 phenotype to an M2-like phenotype. These changes were accompanied by inhibition of the JNK/p38 MAPK–HK2 glycolytic pathway and enhanced PPARγ- and CPT1A/ACSL1-mediated fatty acid oxidation (Li et al., 2025b). Although these findings link IPA-induced macrophage metabolic reprogramming to PPARγ-related signaling, the absence of specific pharmacological or genetic interference targeting PPARγ precludes determining whether PPARγ is essential for these effects. It also remains unclear whether IPA acts directly on PPARγ.

Naringin is a flavonoid glycoside derived from grapefruit, lime, and citrus seeds that possesses anti-inflammatory and antioxidant properties. Cao et al. evaluated naringin using a 2.5% DSS-induced acute UC model in C57BL/6 mice and an LPS-stimulated RAW264.7 macrophage model. Naringin (at *in vivo* doses of 25,50,100 mg/kg/day; *in vitro* concentration of 20 μM) alleviated DAI scores, colonic shortening, and histopathological damage; reduced levels of TNF-α, IL-1β, and IL-6; and restored ZO-1 expression. These effects were accompanied by enhanced PPARγ-related signaling and inhibition of NF-κB, MAPK (p38/ERK/JNK), and NLRP3 inflammasome activation. Importantly, GW9662 attenuated the anti-inflammatory effects of naringin, supporting a functional contribution of PPARγ to the observed responses (Cao et al., 2018). However, pharmacological antagonism alone does not prove direct receptor binding, and due to the lack of gene disruption and direct binding experiments, this conclusion only demonstrates functional involvement rather than direct targeting of PPARγ.

### 5.3 PPARγ/Nrf2 signaling pathway

Nrf2 is a key transcription factor that maintains cellular redox homeostasis; under resting conditions, it is primarily regulated by Keap1-mediated ubiquitination and degradation; Upon the onset of oxidative stress, Nrf2 dissociates from Keap1 and translocates into the nucleus, where it binds to the antioxidant response element (ARE) to induce the transcription of antioxidant and cytoprotective genes such as HO-1, NQO1, and GCLC, thereby limiting the accumulation of ROS and oxidative damage (Bae et al., 2024). In UC, persistent inflammation leads to excessive ROS production and redox imbalance in the intestinal mucosa; insufficient Nrf2 defense capacity can further promote NF-κB inflammatory signaling activation, cytokine release, and disruption of the mucosal barrier. Therefore, Nrf2 serves as a critical regulatory node linking oxidative stress and intestinal inflammation (Zhang et al., 2024). Notably, there is a functional interplay between PPARγ and Nrf2: PPARγ activation enhances Nrf2-mediated antioxidant defense, promotes the expression of downstream effector molecules such as HO-1, and reduces ROS and lipid peroxidation levels; at the same time, Nrf2 activation can suppress NF-κB-mediated inflammatory transcription, thereby reducing the expression of pro-inflammatory mediators such as TNF-α, IL-1β, and IL-6, forming a PPARγ/Nrf2-mediated antioxidant–anti-inflammatory synergistic network. This signaling network also mitigates oxidative stress-induced damage to tight junctions, maintains the expression of barrier proteins such as ZO-1 and Occludin, reduces intestinal mucosal permeability, and limits the continuous stimulation of the mucosal immune system by luminal antigens (Peng et al., 2023). Therefore, decreased PPARγ activity in UC may weaken Nrf2-mediated antioxidant defenses, leading to a mutually reinforcing cycle of ROS accumulation, NF-κB activation, and barrier damage; restoring PPARγ/Nrf2 signaling homeostasis may be a key mechanism for suppressing the sustained amplification of UC inflammation and promoting mucosal barrier repair.

Moringin (MOR) is an isothiocyanate bioactive compound derived from *Moringa oleifera* Lam. (Moringaceae) that possesses anti-inflammatory and antioxidant properties. Zhang et al. evaluated the effects of MOR using a 2.5% DSS-induced colitis model in C57BL/6 mice and an LPS-stimulated Caco-2 cell model. MOR improved DAI scores, colonic shortening, and histopathological damage; reduced levels of TNF-α, IL-1β, and IL-6; and restored the expression of ZO-1, occludin, tight junction protein-1, and Muc2. These effects were associated with the modulation of PPARγ-related signaling pathways, the activation of the Nrf2/HO-1 antioxidant pathway, the inhibition of the NF-κB signaling pathway, and alterations in the PI3K/AKT/mTOR signaling pathway. Both Nrf2 gene knockout and pharmacological inhibition with ML385 attenuated the protective effects of MOR, providing direct functional evidence for the necessity of Nrf2 in this response (Zhang et al., 2024). In contrast, evidence regarding PPARγ remains insufficient and is primarily based on changes in expression and the pharmacological antagonism of GW9662. Therefore, this study provides stronger causal support for the involvement of Nrf2 than for direct targeting of PPARγ, the latter of which still requires confirmation through genetic and target-binding approaches.

### 5.4 PPARγ/STAT signaling pathway

STAT is a family of transcription factors that mediate both cytoplasmic signal transduction and nuclear transcriptional regulation. This family includes members such as STAT1 through STAT6, which, in response to cytokine stimulation, undergo phosphorylation, dimerization, and translocation into the nucleus, thereby regulating the expression of genes related to inflammation, immune differentiation, and cell survival. In UC, the abnormal activation of STAT1 and STAT3 is closely associated with persistent mucosal inflammation and T-cell immune dysregulation. Inflammatory mediators such as IL-6 can drive STAT3 phosphorylation, promote RORγt expression and Th17 differentiation, and increase IL-17A release; meanwhile, sustained amplification of STAT signaling can also amplify pro-inflammatory transcriptional programs, exacerbating colonic mucosal damage (Hu, 2025). As a key nuclear receptor transcription factor, PPARγ exerts negative regulation on STAT-related inflammatory and immune signaling: PPARγ activation inhibits the STAT3/HIF-1α axis, restricts Th17 cell differentiation, and simultaneously promotes the maintenance of the Treg immune phenotype and anti-inflammatory mediators such as IL-10 and TGF-β, thereby restoring the disrupted Treg/Th17 homeostasis in UC (Zhao et al., 2021). Furthermore, PPARγ can indirectly limit the activation of pro-inflammatory transcription factors such as STAT1 by reducing MAPK-mediated abnormal phosphorylation, thereby decreasing the expression of TNF-α, IL-6, and iNOS. Therefore, PPARγ′s regulation of STAT signaling is not limited to a single inflammatory factor; rather, by coordinating STAT3-mediated adaptive immune differentiation with STAT1-related pro-inflammatory transcription, it suppresses the amplification of inflammatory cascades and maintains intestinal mucosal immune homeostasis. This suggests that the PPARγ/STAT signaling network may serve as a critical molecular link between inflammation and immune dysregulation in UC.

Paris polyphylla extract (EPP) was evaluated by He et al. in acute and chronic DSS-induced UC models in C57BL/6 mice. EPP (50,100 mg/kg/day) reduced DAI scores and histopathological injury, decreased IL-6, IL-1β, and TNF-α levels, restored ZO-1, occludin, and claudin-1 expression, and partially corrected the Treg/Th17 imbalance. These effects were accompanied by increased PPARγ expression and suppression of STAT3/HIF-1α signaling. Importantly, the PPARγ antagonist GW9662 attenuated the intestinal protective effects of EPP, supporting the functional involvement of PPARγ-related signaling in the observed response (He et al., 2023). However, pharmacological antagonism does not establish direct target engagement, and the absence of PPARγ genetic perturbation limits causal inference. More importantly, EPP is a multicomponent extract for which the constituent(s) responsible for PPARγ-related activity were not identified; therefore, direct attribution of PPARγ modulation to EPP as a whole should be interpreted cautiously.

Shi et al. evaluated Alliin using a 2% DSS-induced colitis model in ICR mice and an LPS-stimulated RAW264.7 macrophage model. Alliin alleviated weight loss, DAI scores, and colonic damage, and reduced the levels of MPO, MDA, NO, TNF-α, IL-1β, IL-6, and iNOS. Molecular analysis revealed reduced phosphorylation levels of ERK1/2, JNK, and p38 MAPK, along with alterations in PPARγ-related signaling pathways and inhibition of the NF-κB/AP-1/STAT1 inflammatory signaling pathway (Shi et al., 2017). However, the study did not include any experiments with PPARγ-specific antagonists, gene disruption experiments, receptor function assays, or direct binding assays. Therefore, while the study supports an association between Alliin treatment and PPARγ-related molecular changes, it does not establish PPARγ as a functionally essential or direct molecular target of Alliin.

### 5.5 PPARγ/AMPK signaling pathway

AMPK is a serine/threonine protein kinase composed of an α catalytic subunit and β and γ regulatory subunits; it also serves as a key signaling hub linking cellular stress sensing to the regulation of inflammation and immunity (Kunanusornchai et al., 2016). In UC, abnormal AMPK activity may contribute to intestinal immune dysregulation and mucosal barrier damage; its activation can influence macrophage polarization and adaptive immune responses by forming a synergistic regulatory axis with PPARγ (Li et al., 2025b). On the one hand, enhanced AMPK phosphorylation promotes PPARγ activity and expression, inhibits the shift of macrophages toward the M1 phenotype, reduces the release of pro-inflammatory mediators such as iNOS and TNF-α, and simultaneously promotes the expression of M2 markers such as Arg1 and CD206, thereby alleviating local inflammatory responses; On the other hand, the PPARγ/AMPK signaling pathway can influence the differentiation balance between Th17 and Treg cells by regulating ACC1-related metabolic processes, thereby suppressing excessive Th17 immune responses and promoting Treg-mediated immune tolerance (Xu et al., 2017). Furthermore, AMPK and PPARγ signaling are involved in maintaining the expression of tight junction proteins such as Occludin and ZO-1, as well as the integrity of the intestinal mucosal barrier. Therefore, AMPK dysfunction may promote the persistence of UC inflammation through the “AMPK-PPARγ axis,” while the restoration of its activity helps to reestablish intestinal immune homeostasis and mucosal barrier function.

Luteolin is a naturally occurring flavonoid found in *Lonicera japonica* Thunb. (Caprifoliaceae). and possesses anti-inflammatory and immunomodulatory effects. Yang et al. evaluated the effects of luteolin using a DSS-induced model of UC in BALB/c mice and an LPS-stimulated RAW264.7 macrophage model. Luteolin alleviated weight loss, DAI scores, colonic shortening, and histopathological damage; improved intestinal barrier function; reduced levels of TNF-α, IL-6, and IL-1β; and promoted a shift in the macrophage phenotype from M1-like to M2-like. These changes were accompanied by enhanced AMPK/PPARγ-related signaling pathways, decreased iNOS expression, and increased Arg1 and CD206 expression. Compound C and GW9662 partially attenuated these effects, supporting the functional involvement of AMPK and PPARγ-related signaling pathways (Yang et al., 2025). However, these pharmacological data do not prove that luteolin acts directly or specifically on PPARγ. Given the reported PAINS/assay interference issues associated with luteolin, this distinction is particularly important: nonspecific interactions or assay-dependent interference may complicate the interpretation of *in vitro* isolated target-related effects. Therefore, before drawing conclusions regarding a mechanism that directly targets PPARγ, direct binding assays, PPARγ transcriptional activity assays, gene disruption studies, and orthogonal reverse screening for non-specific interference must be conducted.

Extracts of Gleditsia sinensis Lam. Thorn (EGST) are natural plant extracts derived from *Gleditsia sinensis* Lam. (Fabaceae) that possess anti-inflammatory and immunomodulatory effects. Liu et al. used a 3% DSS-induced colitis model in C57BL/6 mice to evaluate the efficacy of EGST (80, 160,320 mg/kg). EGST attenuated weight loss, DAI scores, colonic shortening, and histopathological damage; reduced levels of TNF-α, IL-6, and IL-1β; increased IL-10 levels; restored goblet cell numbers and the expression of ZO-1 and claudin; and was associated with changes in the gut microbiota and metabolic profiles. Transcriptomic and Western blot analyses further confirmed alterations in PPARγ/AMPK and NF-κB-related signaling pathways (Liu X. et al., 2025). However, transcriptomic enrichment analysis and changes in protein expression provided only pathway-level correlations, rather than evidence of PPARγ dependence. Since EGST is a multicomponent extract, neither the specific active components nor a PPARγ-specific functional relationship has been established.

Madecassic acid is a pentacyclic triterpenoid associated with the constituents of *Centella asiatica* (L.) Urb. (Apiaceae). Xu et al. used a DSS-induced model of UC in C57BL/6 mice to evaluate the efficacy of madecassic acid (12.5.25 mg/kg), with cyclosporine A (25 mg/kg) serving as the positive control. Following treatment, DAI scores decreased, colon length was restored, MPO activity and histopathological damage were reduced, and the Th17/Treg imbalance was improved. Mechanistically, these effects were associated with PPARγ activation, increased AMPK phosphorylation, decreased ACC1 expression, inhibition of RORγt and Th17-associated cytokines, and increased Foxp3 and IL-10 expression. Importantly, complementary pharmacological and genetic approaches further strengthened the evidence for these mechanisms: GW9662 and T0070907 attenuated this response; PPARγ siRNA confirmed that the effect was PPARγ-dependent; and TR-FRET, EMSA, and PPARγ reporter assays provided additional evidence for receptor binding and functional activation (Xu et al., 2017). Compared with studies relying solely on PPARγ expression or pharmacological antagonism, this multimodal evidence provides stronger support for a PPARγ-dependent mechanism. Nevertheless, the existing evidence primarily stems from preclinical studies and is mainly based on acute DSS models; to establish the physiological and translational relevance of this mechanism, it remains necessary to develop cell-specific *in vivo* genetic models, perform pharmacokinetic characterization, and conduct long-term safety evaluations.

### 5.6 PPARγ/SIRT signaling pathway

SIRTs are a highly conserved family of NAD^+^-dependent deacetylases comprising SIRT1–SIRT7. Among them, SIRT1 is the most extensively studied member; it participates in the regulation of inflammatory responses, oxidative stress, cell survival, and energy homeostasis by deacetylating transcription factors and metabolic regulatory proteins, serving as a crucial molecular hub linking cellular metabolic status to immune and inflammatory responses (Carafa et al., 2016). In the pathological progression of UC, persistent inflammatory stimulation and oxidative stress may be accompanied by a decrease in SIRT1 expression or activity in colonic tissue, thereby weakening its negative regulation of pro-inflammatory transcriptional programs; Conversely, enhancing SIRT1 activity can limit the expression of pro-inflammatory mediators such as TNF-α and IL-6 by deacetylating inflammation-related transcription factors like NF-κB p65, while simultaneously improving cellular antioxidant defenses, thereby inhibiting the amplification of inflammatory cascades and oxidative damage. Notably, a functional synergy exists between SIRT1 and PPARγ, with both jointly participating in the regulation of intestinal mucosal inflammation and redox homeostasis; Activation of the PPARγ/SIRT1 axis enhances anti-inflammatory transcriptional programs and promotes the restoration of endogenous antioxidant systems such as SOD and GSH, reducing the accumulation of lipid peroxidation products like MDA, thereby alleviating inflammation-mediated damage to colonic tissue (Raslan et al., 2026). Furthermore, in UC models, the expression of both PPARγ and SIRT1 is simultaneously reduced; restoration of this signaling axis is associated with decreased levels of TNF-α, IL-6, and calmodulin, increased levels of IL-10, and alleviated oxidative stress. This suggests that PPARγ/SIRT1-mediated synergistic regulation of inflammation and oxidative stress may be a key mechanism for maintaining colonic mucosal homeostasis and limiting the progression of UC.

Extracts from the aerial parts of *Crassula tetragona* L. (Crassulaceae; CT) are rich in bioactive constituents, including flavonoids and phenolic acids. Raslan et al. evaluated a CT1 and a 70% CT2 in an acetic acid-induced rat colitis model over a 7-day period at doses of 200 and 400 mg/kg/day, respectively, without a positive drug control. HPLC–ESI–MS/MS analysis identified naringin, gallic acid, and quercetin as the major components of CT2. Both extracts alleviated colonic injury, reduced levels of CRP, calprotectin, TNF-α, and IL-6, increased IL-10 levels, and improved oxidative stress markers, including SOD, GSH, and MDA. These effects were accompanied by increased tissue expression levels of SIRT1 and PPARγ (Raslan et al., 2026). However, the concurrent increase in SIRT1 and PPARγ expression alone is insufficient to establish a functional SIRT1/PPARγ signaling mechanism. In the absence of pathway-specific pharmacological blockade, genetic disruption, cellular functional assays, or direct target binding assays, the existing evidence remains merely correlational. Furthermore, since CT1 and CT2 are multicomponent extracts, the specific constituents responsible for these molecular changes remain unclear.

### 5.7 PPARγ/mTOR signaling pathway

The mammalian target of rapamycin (mTOR) is a highly conserved serine/threonine protein kinase that integrates signals from nutrients, growth factors, and cellular energy status, and participates in the regulation of cellular metabolism, proliferation, and immune responses through two complexes: mTORC1 and mTORC2 (Goul et al., 2023). In UC, abnormal mTOR signaling is closely associated with the immunometabolic reprogramming of macrophages and an imbalance in the inflammatory microenvironment. Activation of mTORC1 promotes the stabilization of HIF-1α and its transcriptional activity, enhances glycolytic pathways such as HK2, PKM2, and LDHA, shifts macrophage metabolism toward aerobic glycolysis, and promotes M1 polarization and the production of pro-inflammatory mediators such as TNF-α, IL-1β, and IL-6, thereby sustaining mucosal inflammation; Conversely, inhibition of the mTORC1/HIF-1α axis can limit glycolysis and the M1 phenotype, thereby alleviating inflammatory damage (Wu et al., 2021). In contrast, mTORC2 is associated with PPARγ-mediated fatty acid oxidation and M2 polarization, promoting the formation of an anti-inflammatory phenotype; thus, the differential regulation of mTORC1 and mTORC2 jointly determines the metabolic and functional states of macrophages. Furthermore, upregulation of PPARγ negatively regulates mTOR/HIF-1α signaling, promotes mitochondrial oxidative phosphorylation and M2 polarization, thereby reducing the release of pro-inflammatory factors and improving colonic mucosal damage (Hu et al., 2024). Consequently, mTOR is not merely a pro-inflammatory signaling node but rather a critical hub linking PPARγ, HIF-1α, immunometabolism, and macrophage polarization; the functional balance among its different complexes may determine the persistence or resolution of inflammation in UC.

Dioscin is a steroidal saponin found in *Dioscorea nipponica* Makino (Dioscoreaceae) that possesses anti-inflammatory and immunomodulatory effects. Wu et al. evaluated Dioscin in a 3% DSS-induced UC model in BALB/c mice, as well as in LPS/IFN-γ-induced M1 polarization and IL-4-induced M2 polarization models established using RAW264.7 macrophages. Diocsin improved DAI scores, reduced colonic shortening, and alleviated histopathological damage; it also decreased levels of MPO, TNF-α, IL-6, and IFN-γ, and promoted a shift in macrophage polarization from the M1 to the M2 phenotype. These effects were associated with the inhibition of mTORC1/HIF-1α-mediated glycolysis and the enhancement of fatty acid metabolism related to mTORC2/PPARγ. Pharmacological interventions targeting the mTOR signaling pathway using L-leucine and JR-AB2-011 attenuated the protective effects of dioxin, supporting the functional role of differential regulation of mTORC1 and mTORC2 (Wu et al., 2021). However, since these interventions targeted mTOR rather than PPARγ itself, they could not demonstrate that Dioscin’s effects are PPARγ-dependent, nor could they prove that dioxin directly activates PPARγ. Therefore, pharmacological or genetic interference targeting PPARγ, along with receptor functional assays, is needed to distinguish between PPARγ-associated downstream associations and PPARγ-dependent mechanisms.

Berberine (BBR) is an isoquinoline alkaloid found in medicinal plants including *Coptis chinensis* Franch. (Ranunculaceae) and *Phellodendron chinense* C.K.Schneid. (Rutaceae), and possesses anti-inflammatory and immunomodulatory properties. He et al. evaluated the effects of BBR using a 2.3% DSS-induced colitis model in C57BL/6J mice and an IL-4/IL-13-induced M2-polarized RAW264.7 model. Using 5-ASA (200 mg/kg/day) as a positive control, BBR(50,100 mg/kg/day) alleviated weight loss, colonic shortening, and histopathological damage; reduced the expression of IL-6, IL-1β, and TNF-α; and promoted M2-like macrophage polarization and oxidative phosphorylation. These effects were accompanied by the enhancement of PPARγ-related signaling pathways and the inhibition of the mTOR/HIF-1α signaling pathway. GW9662 attenuated the metabolic and phenotypic effects of BBR, thereby providing pharmacological evidence that PPARγ plays a functional role in BBR-induced macrophage metabolic reprogramming (He et al., 2026). However, pharmacological antagonism alone is insufficient to establish a direct compound-receptor interaction, and due to the lack of PPARγ gene disruption experiments and direct target binding assays, it is not possible to conclude that PPARγ is a direct molecular target of BBR.

## 6 Discussion

Current research indicates that PPARγ is not a single anti-inflammatory target, but rather a key molecular hub linking intestinal inflammatory responses, immune regulation, gut microbiota metabolism, and mucosal barrier homeostasis (Fang et al., 2021). In UC, reduced PPARγ expression or transcriptional activity weakens its negative regulation of pro-inflammatory signaling pathways such as NF-κB, MAPK, and STAT, and is closely associated with pathological changes including Th17/Treg imbalance, abnormal M1/M2 polarization of macrophages, disruption of the gut microbiota and its metabolism, and damage to tight junctions (Bashiri et al., 2026). Previous studies have shown that various natural products—including flavonoids, phenolics, terpenoids, alkaloids, coumarins, polysaccharides, and plant extracts—can modulate PPARγ and its associated signaling networks to varying degrees, accompanied by reduced inflammatory responses, restored immune homeostasis, and improved mucosal barrier function (Zhou Q. et al., 2023). This suggests that PPARγ may serve as a key molecular node through which natural products exert their multi-target anti-UC effects. However, existing evidence still primarily stems from cellular and animal experiments, with most studies relying on changes in PPARγ expression and its downstream signaling responses as the basis for their mechanisms. Therefore, current research largely supports the notion that PPARγ “participates” in the intestinal protective effects of natural products, but is insufficient to universally prove that it is a direct and specific target of these natural products. Future research will need to further refine the evidence base by addressing issues such as direct targeting versus function-dependent mechanisms, the specificity of mechanisms to cellular and disease contexts, *in vivo* pharmacological characteristics, and clinical translation.

First, the evaluation of direct targeting of PPARγ and the functional necessity of such targeting should be strengthened to avoid defining a compound as a direct target solely based on molecular docking, increased PPARγ expression, or changes in downstream signaling (Wang et al., 2024). Some natural compounds, such as polyphenols, flavonoids, and quinones, may exhibit characteristics such as nonspecific protein binding, colloidal aggregation, redox reactions, metal chelation, or membrane disruption, which can interfere with *in vitro* experimental results and increase the risk of false positives. For compounds with potential PAINS structural alerts, such as luteolin and emodin, relying solely on molecular docking or a single *in vitro* experiment is insufficient to fully demonstrate the specificity of their action on PPARγ; for complex plant extracts rich in polyphenols, flavonoids, and other components, their chemical composition and potential targets are even more complex, and the uncertainty in target attribution is more pronounced (Spiegler, 2026). It should be noted that a PAINS structural alert does not necessarily imply that the pharmacological activity is a false positive; rather, it indicates that evidence regarding the relevant targets requires more rigorous evaluation. In the future, orthogonal experiments based on different detection principles, as well as reverse screening strategies such as aggregation and redox reactions, can be employed to rule out nonspecific interference (Tan et al., 2024). These approaches should be combined with direct binding assays such as SPR, MST, or ITC, PPARγ transcriptional activity assays, specific pharmacological blockade, and genetic interventions to gradually establish a complete chain of evidence:“exclusion of nonspecific interference-direct target binding-receptor function regulation-PPARγ dependence-in vivo efficacy evaluation” (Atz et al., 2024). For complex plant extracts, chemical characterization, activity-guided isolation, and back-filling experiments with individual compounds or combinations of components should also be integrated to further clarify the key molecular basis mediating PPARγ regulation.

Second, the cell-specificity of PPARγ′s actions and the robustness of its mechanisms across different UC pathological contexts should be further clarified. PPARγ exhibits distinct transcriptional regulatory networks and biological functions in intestinal epithelial cells, macrophages, dendritic cells, and T lymphocytes; however, most existing studies are based on analyses of whole colon tissue or single cell lines, making it difficult to distinguish the actual contributions of PPARγ from different cell sources to the pharmacological effects of natural products (Bashiri et al., 2026). In the future, intestinal epithelial cell-, myeloid cell-, or T-cell-specific PPARγ knockout models could be introduced and combined with techniques such as single-cell transcriptomics and spatio-omics to identify the key effector cells involved in the action of natural products and their downstream regulatory networks. At the same time, current research relies heavily on the acute DSS colitis model, which fails to adequately reflect the characteristics of human UC, such as chronic course, recurrent episodes, and immunological heterogeneity. Therefore, based on chronic or recurrent DSS models, experimental colitis models with different pathological mechanisms—such as TNBS and T-cell adoptive transfer—could be integrated, and research systems more closely resembling human disease characteristics, such as patient-derived colonic organoids, could be appropriately introduced for cross-evaluation. Only when PPARγ-dependent effects are repeatedly validated across different cell types and disease models can the causal strength, robustness, and biological extrapolation value of these mechanisms be further enhanced.

The pharmacokinetics, bioavailability, dose–response relationships, and long-term safety of natural products are also key issues that must be addressed during the translation of mechanistic research into clinical applications. *In vitro* PPARγ modulatory activity does not necessarily imply that corresponding effective exposure levels can be achieved in colonic tissue following oral administration. Some natural compounds also suffer from poor water solubility, limited intestinal absorption, insufficient *in vivo* stability, or rapid metabolic clearance; moreover, many current studies have yet to establish a quantitative relationship between drug exposure, the degree of PPARγ modulation, and anti-colitis effects. Future studies should systematically evaluate the absorption, distribution, metabolism, and excretion characteristics of candidate compounds, simultaneously measuring the levels of the parent compound and active metabolites in plasma, colonic tissue, and, when necessary, intestinal contents. Furthermore, a correlation analysis should be conducted between actual tissue exposure, the degree of PPARγ modulation, and anti-UC effects. Through standardized dose–response and time–response studies, the effective dose, duration of action, and appropriate therapeutic window should be determined. Given that PPARγ is extensively involved in lipid metabolism, glucose homeostasis, and the regulation of adipocyte function, long-term or non-selective activation may also produce extraintestinal metabolic effects; therefore, evaluations of long-term toxicology, major organ function, and systemic metabolic effects should be strengthened. Based on the identification of effective tissue exposure, stable dose-response relationships, and a safety window, strategies such as colon-targeted delivery can be explored to enhance local drug exposure at the site of the lesion and reduce effects on non-target tissues, thereby providing a more robust pharmacological basis for the long-term treatment of UC with natural products.

Finally, the translation of PPARγ-related natural products into clinical research should be advanced based on robust preclinical evidence. Existing cellular and animal studies provide important evidence for elucidating potential mechanisms of action and screening candidate therapeutic molecules; however, they are not yet sufficient to demonstrate clinical efficacy in human UC. Therefore, current discussions regarding clinical translation should be viewed as future research directions rather than as confirmation of existing clinical efficacy. Before candidate natural compounds enter efficacy-based clinical trials, their pharmacokinetics, tolerability, safety, and appropriate dosage range in humans must be thoroughly characterized. On this basis, their therapeutic effects should be evaluated through standardized clinical trials, with comprehensive assessments based on multidimensional objective endpoints, including clinical remission, endoscopic mucosal healing, histological improvement, and fecal calprotectin levels. Overall, future research should gradually shift from focusing on PPARγ expression or signaling relevance to direct targeting and verification of functional causality. While clarifying the basis of the active substance and the necessity of PPARγ function, the robustness of the mechanism should be validated through cell-specific genetic models and multi-type UC models, and key issues such as *in vivo* exposure, dose–response relationships, and long-term safety, ultimately establishing a complete chain of evidence spanning the identification of active compounds, target evaluation, mechanism elucidation, pharmacological evaluation, and clinical translation.

Overall, PPARγ is not only an anti-inflammatory target but also a key molecular hub linking inflammatory transcription, immune cell fate, the gut microbiota, and mucosal barrier homeostasis; this characteristic provides an important theoretical basis for multi-target interventions using natural products to treat ulcerative colitis. As these issues are gradually resolved, it is anticipated that drug candidates with well-defined PPARγ regulatory mechanisms, good colonic exposure, and high safety profiles will be identified through screening of natural products. These drug candidates will offer new pharmacological options for the induction of remission in patients with mild to moderate ulcerative colitis, as well as for long-term maintenance therapy in patients with chronic, recurrent disease, thereby advancing the treatment of ulcerative colitis from mere inflammation suppression toward the restoration of mucosal homeostasis and immune-microbiome balance.
